# Supplementary material for: Self-supervised learning enables 3D digital subtraction angiography reconstruction from ultra-sparse 2D projection views: A multicenter study
Source: Cell Rep Med. 2022 Oct 7;3(10):100775. doi: 10.1016/j.xcrm.2022.100775 (PMC9589028; doi:10.1016/j.xcrm.2022.100775)
Supplement: Document S1. Figures S1–S6 and Tables S1–S11 and S14 [file mmc1.pdf]

Cell Reports Medicine, Volume *3*

Supplemental information

Self-supervised learning enables 3D digital subtraction angiography reconstruction from ultra-sparse 2D projection views: A multicenter study

Huangxuan Zhao, Zhenghong Zhou, Feihong Wu, Dongqiao Xiang, Hui Zhao, Wei Zhang, Lin Li, Zhong Li, Jia Huang, Hongyao Hu, Chengbo Liu, Tao Wang, Wenyu Liu, Jinqiang Ma, Fan Yang, Xinggang Wang, and Chuansheng Zheng

Supplementary Figures

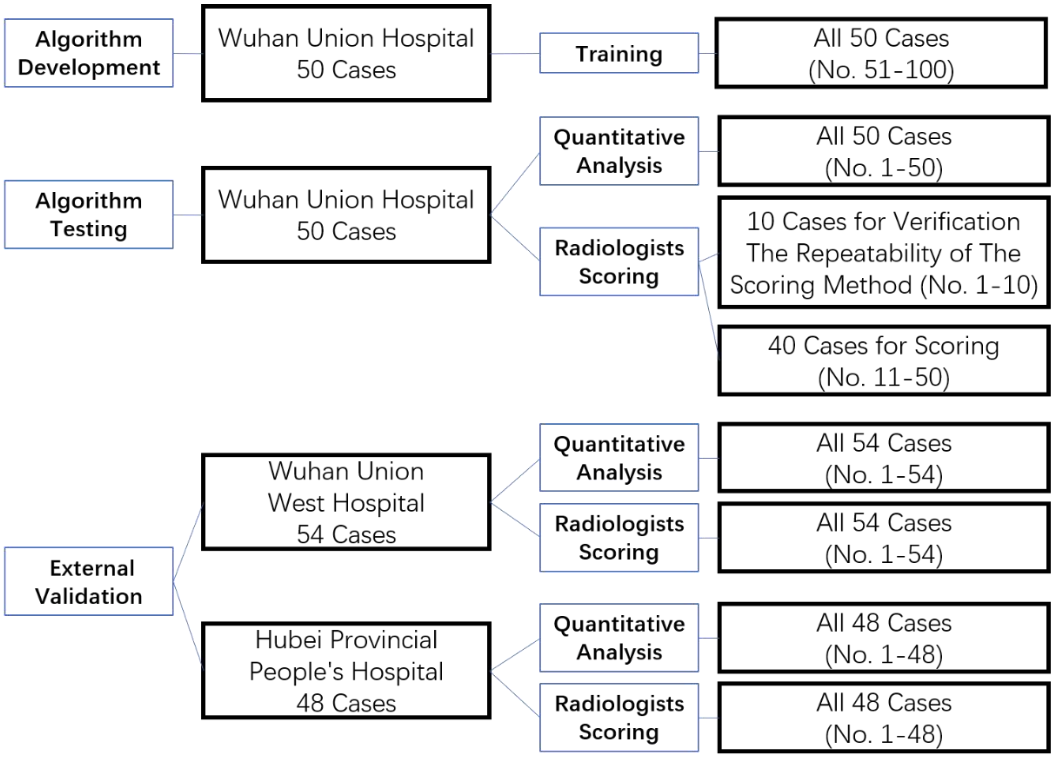

Figure S1. Flowchart outlining the data acquisition methods and the divisions made. Related to STAR METHODS.

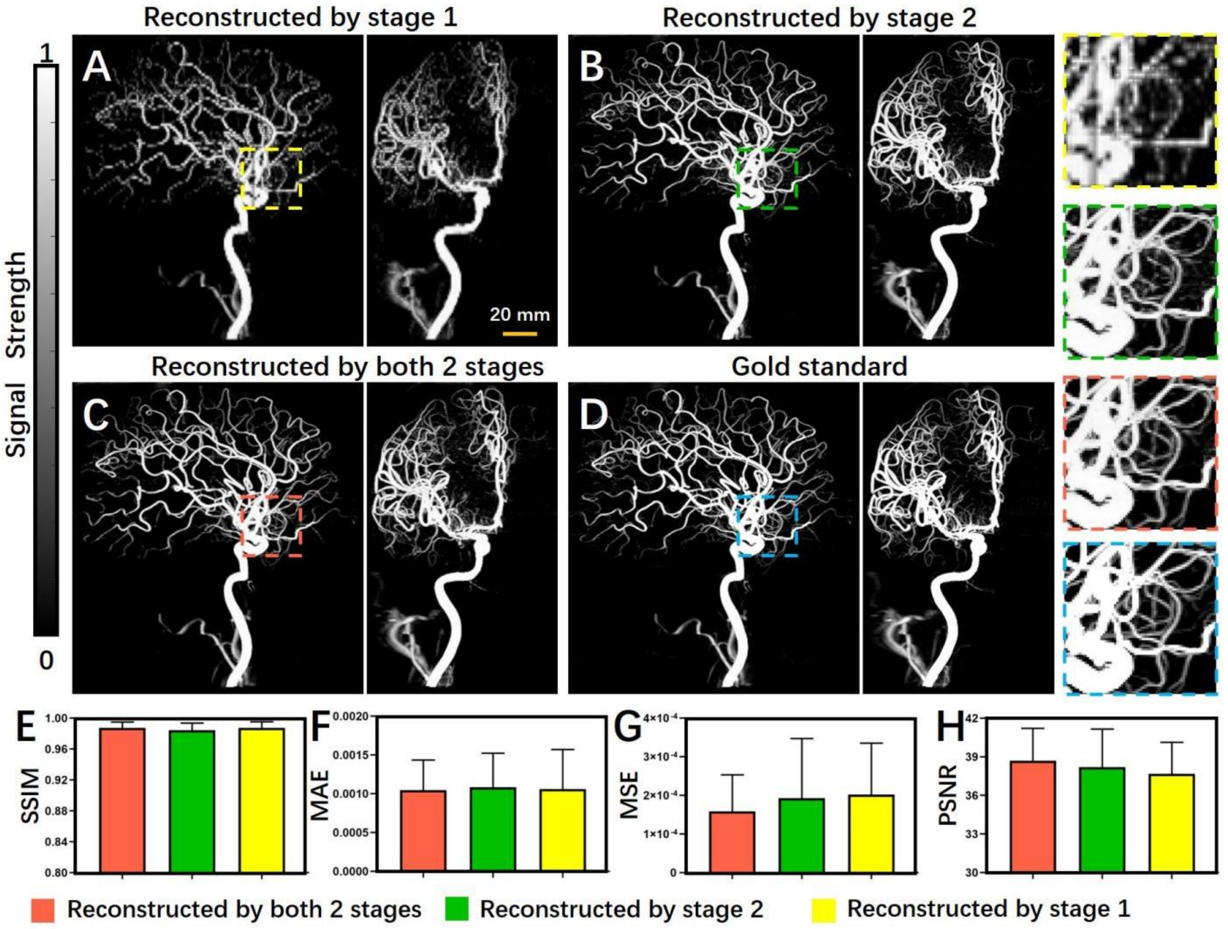

Figure S2. Comparison of reconstruction results. (A)–(D) Reconstruction images obtained from stage 1, stage 2, both 2 stages, and the gold standard. (E)–(H) Results obtained by quantitatively evaluating 50 cases of Institution I using SSIM/MAE/MSE/PSNR. Related to STAR METHODS.

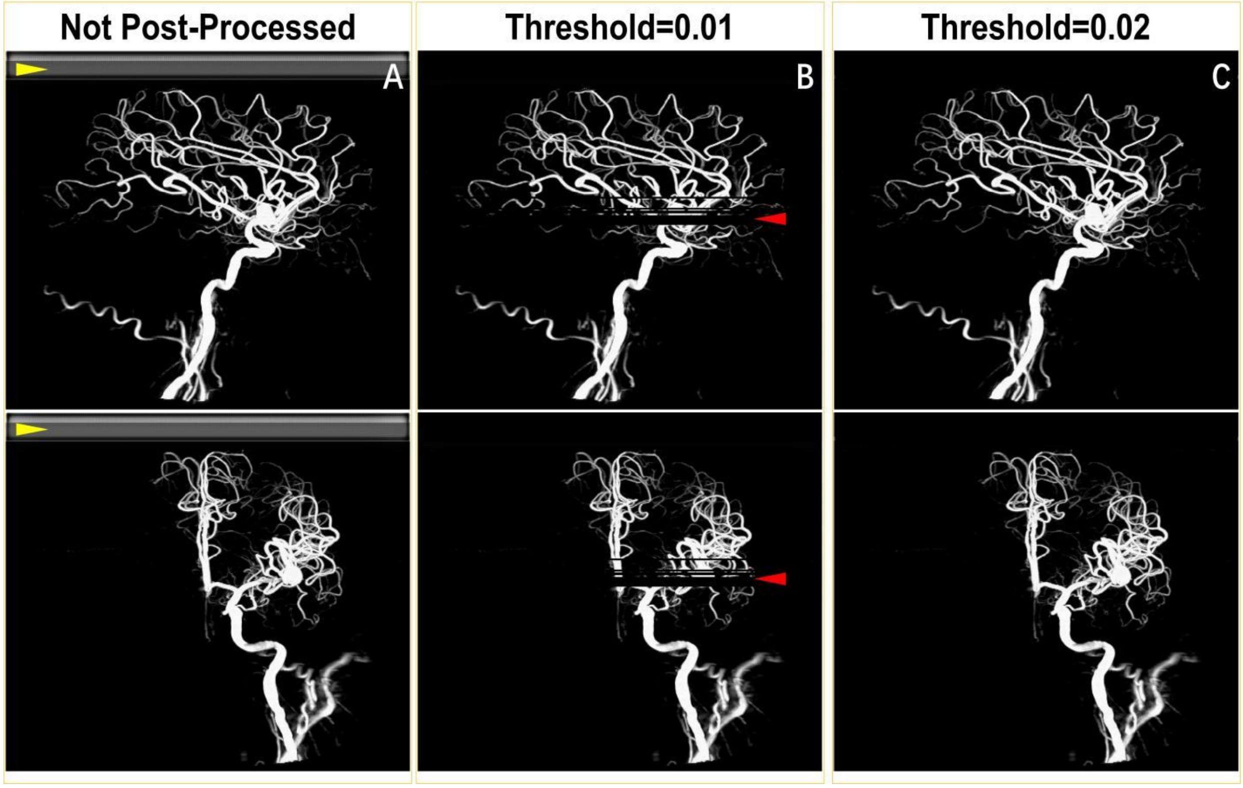

Figure S3. Reconstructed images. (A) Post-processed method not followed. (B) – (C) Post-processing method was followed (B. threshold=0.01 and C. threshold=0.02). Related to STAR METHODS.

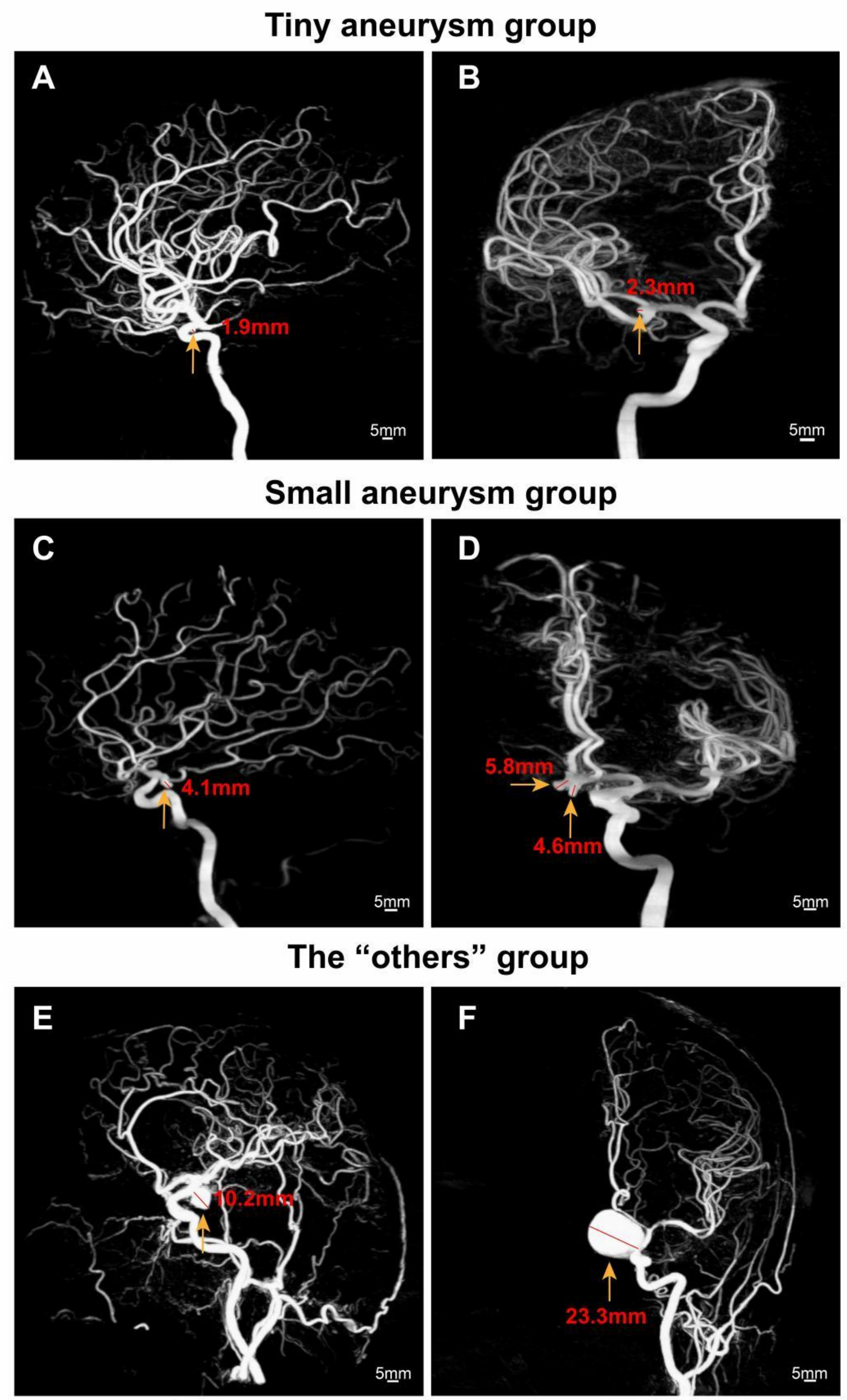

Figure S4. Random cases selected for presentation. (A) Tiny intracranial aneurysm (long diameter: 1.9 mm) located in the C6 segment. (B) Tiny aneurysm (long diameter: 2.3 mm) present at the bifurcation of the middle cerebral artery. (C) Small aneurysm (long

diameter: 4.1 mm) at the origin of the posterior cerebral artery. (D) Two aneurysms (long diameters: 5.8 mm and 4.6 mm, respectively) originating from the anterior communicating artery. (E) Significantly large intracranial aneurysm (long diameter: 10.2 mm) present in the C6 segment. (F) Large intracranial aneurysm (long diameter: 23.3 mm) originating from the C6 segment, and the A1 segment of the anterior cerebral artery was pushed upward. Related to STAR METHODS.

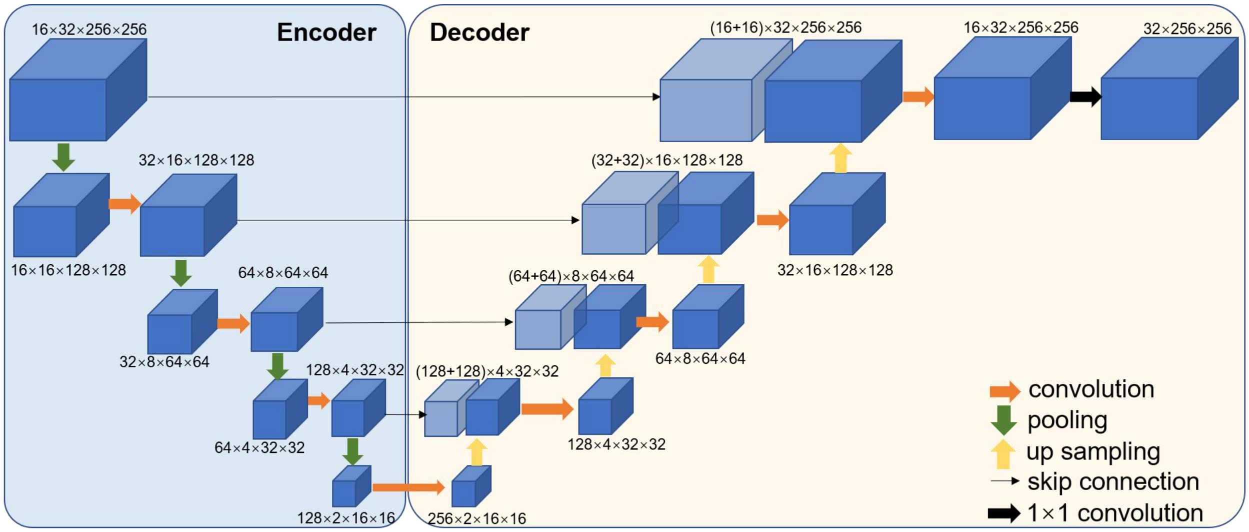

Figure S5. Encoding and decoding processes followed during the low-resolution reconstruction stage. Related to Figure 2.

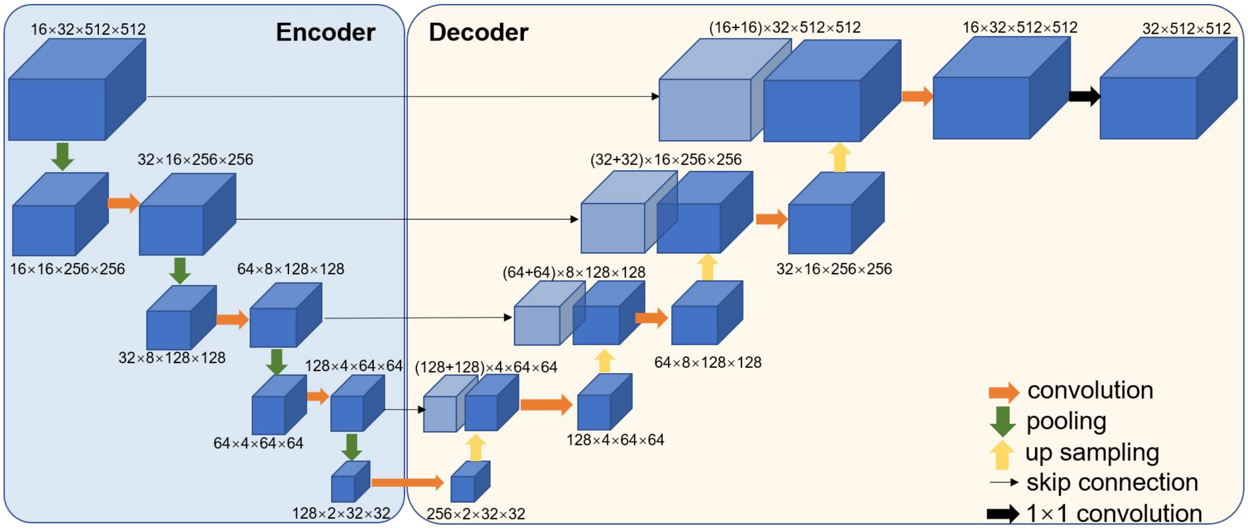

Figure S6. Encoding and decoding processes followed during the high-resolution reconstruction stage. Related to Figure 2.

Supplementary Tables

Table S1. Angles used for 4/6/8/10/12 projection views. Related to Figure 2.

Input Projections	Used Angles
4	45°/90°/135°/180°
6	30°/60°/90°/120°/150°/180°
8	22.5°/45°/67.5°/90°/112.5°/135°/157.5°/180°
10	18°/36°/54°/72°/90°/108°/126°/144°/162°/180°
12	15°/30°/45°/60°/75°/90°/105°/120°/135°/150°/165°/180°

Table S2. SSIM, PSNR, MSE, and MAE values corresponding to the reconstruction results under conditions of different cover angles of inputs with 8 projection views. Related to STAR METHODS.

Cover angles	SSIM	PSNR	MSE	MAE
45°	.969±.018	32.45±1.34	.000594±.000171	.002150±.000651
90°	.970±.018	35.17±1.59	.000324±.000113	.001714±.000610
180°	.982±.019	39.74±2.25	.000121±.000065	.000830±.000621

Table S3. SSIM, PSNR, MSE, and MAE values corresponding to the reconstruction results under conditions of different training data sizes. Related to STAR METHODS.

Training data sizes	SSIM	PSNR	MSE	MAE
50 cases	.982±.019	39.74±2.25	.000121±.000065	.000830±.000621
75 cases	.981±.016	40.21±2.17	.000108±.000057	.001211±.000500
100 cases	.980±.021	39.56±2.25	.000127±.000070	.001183±.000700

Table S4. SSIM/PSNR/MSE/MAE values corresponding to the multicenter 3D reconstruction images (4/6/8/10/12 projections were used). The best one is marked in red. Related to Figure 3 and Figure 4.

Institution	Input Projections	SSIM	PSNR	MSE	MAE
I	4	.954±.018	34.35±2.03	.000405±.000173	.002488±.000721
	6	.972±.014	37.49±2.42	.000206±.000111	.001771±.000524
	8	.987±.007	38.69±2.49	.000158±.000094	.001047±.000385
	10	.984±.008	39.30±2.54	.000139±.000085	.001078±.000453
	12	.984±.009	39.95±2.51	.000119±.000073	.001069±.000487
II	4	.959 ± .013	35.27 ± 1.56	.000314 ± .000095	.002160 ± .000427
	6	.977 ± .008	38.86 ± 1.76	.000140 ± .000052	.001472 ± .000270
	8	.989 ± .006	40.06 ± 1.66	.000106 ± .000038	.000855 ± .000223
	10	.987 ± .007	40.72 ± 1.59	.000090 ± .000032	.000843 ± .000249
	12	.988 ± .006	41.43 ± 1.55	.000077 ± .000027	.000805 ± .000239
III	4	.933 ± .022	36.55 ± 1.91	.000243 ± .000103	.002900 ± .000681
	6	.964 ± .022	39.35 ± 2.52	.000137 ± .000080	.001953 ± .000690
	8	.981 ± .016	39.58 ± 2.61	.000132 ± .000081	.001226 ± .000581
	10	.979 ± .017	39.88 ± 2.60	.000123 ± .000075	.001227 ± .000619
	12	.978 ± .017	40.33 ± 2.61	.000111 ± .000067	.001184 ± .000604

Table S5. Scoring method for evaluating 3D reconstruction results (ICA, internal carotid artery; ACA, anterior cerebral artery; MCA, middle cerebral artery). Related to STAR METHODS.

		Score					
		**0**	**1**	**2**	**3**	**4**	**5**
**The overall performance**		Gaps in the image	Severe noise	A lot of noise	Moderate noise	Slight noise	Invisible noise
**Vessel detectability**	**ICA**	Unable to evaluate	Difficulty in identifying vascular occlusion	Difficulty in identifying vascular occlusion stenosis	The vascular boundary of ICA is not clear, but it can be used to identify vascular stenosis	The vascular boundary of ICA is clear, and artifacts exist	The vascular boundary of ICA is clear and devoid of artifacts
	**ACA**	Unclear/not distinguishable	Only the A1 segment is distinguishable	A1 and A2 segments are distinguishable	A1, A2, and A3 segments are distinguishable	/	/
	**MCA**	Unclear/not distinguishable	Only the M1 segment is distinguishable	M1 and M2 segments are distinguishable	M1, M2, and M3 segments are distinguishable	/	/

Table S6. Evaluation results obtained by the two radiologists during the analysis of 30 sets of data. Related to STAR METHODS.

No.	The overall performance		ICA detectability		ACA detectability		MCA detectability	
	R1	R2	R1	R2	R1	R2	R1	R2
**4 Projections**								
**1**	0	0	3	4	0	0	0	0
**2**	0	0	3	3	0	0	0	0
**3**	0	0	4	4	0	0	0	0
**4**	0	0	3	2	3	3	3	3
**5**	3	1	3	3	3	3	3	3
**6**	1	2	3	2	3	3	3	3
**7**	3	3	4	4	3	3	3	3
**8**	0	0	4	4	0	0	0	0
**9**	0	0	4	3	0	0	0	0
**10**	3	3	4	4	3	3	3	3
**6 Projections**								
**1**	0	0	4	4	3	0	3	0
**2**	0	0	4	4	0	0	0	0
**3**	3	1	4	4	3	3	3	3
**4**	3	3	4	3	3	3	3	3
**5**	4	3	4	3	3	3	3	3
**6**	3	3	4	3	3	3	3	3
**7**	4	4	4	4	3	3	3	3
**8**	0	0	4	4	3	3	3	3
**9**	3	2	4	4	3	3	3	3
**10**	4	4	5	4	3	3	3	3
**8 Projections**								
**1**	5	3	5	4	3	3	3	3
**2**	0	2	5	4	3	3	3	3

**3**	4	4	5	4	3	3	3	3
**4**	5	4	5	4	3	3	3	3
**5**	5	3	4	3	3	3	3	3
**6**	5	4	4	4	3	3	3	3
**7**	5	4	5	4	3	3	3	3
**8**	4	3	5	4	3	3	3	3
**9**	3	4	5	4	3	3	3	3
**10**	5	4	5	4	3	3	3	3

Table S7. Scores of the overall image performance obtained by the two radiologists for 40 cases. Related to Table 1.

No.	The overall performance									
	4 Projections		6 Projections		8 Projections		10 Projections		12 Projections	
	R1	R2	R1	R2	R1	R2	R1	R2	R1	R2
**11**	0	0	0	0	5	5	5	5	5	5
**12**	0	0	0	0	0	0	4	4	4	4
**13**	0	0	3	3	4	4	4	4	5	5
**14**	0	0	3	3	5	5	5	5	5	5
**15**	3	3	4	4	5	5	5	5	5	5
**16**	1	1	3	3	5	5	5	5	5	5
**17**	3	3	4	4	5	5	5	5	5	5
**18**	0	0	0	0	4	4	5	5	5	5
**19**	0	0	3	3	3	3	5	4	5	5
**20**	3	3	4	4	5	5	5	5	5	5
**21**	1	2	2	3	3	4	4	4	5	5
**22**	0	0	3	4	4	4	5	5	5	5
**23**	0	0	0	0	4	4	5	4	5	5
**24**	3	3	2	4	4	4	4	5	5	5
**25**	3	3	4	4	5	5	5	5	5	5
**26**	2	3	3	4	4	5	5	5	5	5
**27**	3	3	4	4	5	5	5	5	5	5
**28**	0	0	2	4	4	4	4	5	5	5
**29**	0	0	0	0	4	4	4	4	5	5
**30**	0	0	1	3	3	4	4	5	5	5
**31**	1	3	3	4	4	5	5	5	5	5
**32**	4	4	4	4	5	5	5	5	5	5
**33**	0	0	0	0	0	0	1	1	3	3
**34**	3	3	3	4	4	4	4	5	5	5
**35**	0	0	3	4	5	5	5	5	5	5
**36**	0	0	0	0	4	4	5	5	5	5

**37**	0	0	3	3	4	4	5	5	5	5
**38**	0	0	0	0	3	3	3	3	4	4
**39**	3	4	4	4	5	4	5	5	5	5
**40**	0	0	3	3	4	4	4	4	5	5
**41**	3	3	4	4	5	5	5	5	5	5
**42**	2	3	4	4	0	5	5	5	5	5
**43**	3	3	3	4	5	5	5	5	5	5
**44**	0	0	0	0	4	4	5	5	5	5
**45**	0	0	0	0	4	4	5	5	5	5
**46**	0	0	3	4	4	5	5	5	5	5
**47**	4	3	5	5	5	5	5	5	5	5
**48**	0	0	3	4	4	4	4	5	5	5
**49**	4	4	4	4	5	5	5	5	5	5
**50**	0	0	3	4	5	4	5	5	5	5

Table S8. Scores obtained by the two radiologists for ICA detectability for 40 data. Related to Table 1.

No.	The ICA detectability									
	4 Projections		6 Projections		8 Projections		10 Projections		12 Projections	
	R1	R2	R1	R2	R1	R2	R1	R2	R1	R2
**11**	3	3	4	4	5	5	5	5	5	5
**12**	3	3	4	4	5	5	5	5	5	5
**13**	4	4	4	4	5	5	5	5	5	5
**14**	3	3	4	4	5	5	5	5	5	5
**15**	3	3	4	4	4	4	5	5	5	5
**16**	3	3	4	4	4	4	4	4	5	5
**17**	4	4	4	4	5	5	5	5	5	5
**18**	4	4	4	4	5	5	5	5	5	5
**19**	4	4	4	4	5	5	5	5	5	5
**20**	4	4	5	5	5	5	5	5	5	5
**21**	3	4	4	5	4	5	5	5	5	5
**22**	2	4	4	5	5	5	5	5	5	5
**23**	4	4	4	5	4	5	5	5	5	5
**24**	4	4	5	5	5	5	5	5	5	5
**25**	4	4	4	5	5	5	5	5	5	5
**26**	3	4	4	5	5	5	5	5	5	5
**27**	4	4	4	4	5	5	5	5	5	5
**28**	4	5	4	5	4	5	5	5	5	5
**29**	0	0	2	3	4	5	5	5	5	5
**30**	1	4	4	5	4	5	5	5	5	5
**31**	3	4	4	5	4	5	5	5	5	5
**32**	4	4	5	5	5	5	5	5	5	5
**33**	0	0	1	1	2	2	4	4	4	4
**34**	4	4	5	5	5	5	5	5	5	5
**35**	4	4	4	5	5	5	5	5	5	5
**36**	0	0	0	0	5	5	5	5	5	5

**37**	3	4	4	4	5	5	5	5	5	5
**38**	0	0	4	4	4	4	5	5	5	5
**39**	4	4	4	4	5	4	5	5	5	5
**40**	1	1	4	4	5	5	5	5	5	5
**41**	2	4	5	5	5	5	5	5	5	5
**42**	3	4	4	5	5	5	5	5	5	5
**43**	4	4	4	5	5	5	5	5	5	5
**44**	0	0	3	3	5	5	5	5	5	5
**45**	0	0	0	0	5	5	5	5	5	5
**46**	0	4	4	5	5	5	5	5	5	5
**47**	4	4	5	5	5	5	5	5	5	5
**48**	3	4	3	5	5	5	5	5	5	5
**49**	4	4	4	5	5	5	5	5	5	5
**50**	3	4	4	4	5	5	5	5	5	5

Table S9. Scores obtained by the two radiologists for ACA detectability for 40 data. Related to Table 1.

No.	The ACA detectability									
	**4 Projections**		**6 Projections**		**8 Projections**		**10 Projections**		**12 Projections**	
	R1	R2	R1	R2	R1	R2	R1	R2	R1	R2
**11**	0	0	3	3	3	3	3	3	3	3
**12**	0	0	0	0	3	3	3	3	3	3
**13**	0	0	3	3	3	3	3	3	3	3
**14**	3	3	3	3	3	3	3	3	3	3
**15**	3	3	3	3	3	3	3	3	3	3
**16**	3	3	3	3	3	3	3	3	3	3
**17**	3	3	3	3	3	3	3	3	3	3
**18**	0	0	3	3	3	3	3	3	3	3
**19**	0	0	3	3	3	3	3	3	3	3
**20**	3	3	3	3	3	3	3	3	3	3
**21**	3	3	3	3	3	3	3	3	3	3
**22**	0	0	3	3	3	3	3	3	3	3
**23**	0	0	0	0	3	3	3	3	3	3
**24**	3	3	3	3	3	3	3	3	3	3
**25**	3	3	3	3	3	3	3	3	3	3
**26**	3	3	3	3	3	3	3	3	3	3
**27**	3	3	3	3	3	3	3	3	3	3
**28**	3	3	3	3	3	3	3	3	3	3
**29**	0	0	0	0	3	3	3	3	3	3
**30**	0	0	3	3	3	3	3	3	3	3
**31**	3	3	3	3	3	3	3	3	3	3
**32**	3	3	3	3	3	3	3	3	3	3
**33**	0	0	0	0	0	0	3	3	3	3
**34**	3	3	3	3	3	3	3	3	3	3
**35**	3	3	3	3	3	3	3	3	3	3
**36**	0	0	0	0	3	3	3	3	3	3

**37**	0	0	3	3	3	3	3	3	3	3
**38**	0	0	3	3	3	3	3	3	3	3
**39**	3	3	3	3	3	3	3	3	3	3
**40**	0	0	3	3	3	3	3	3	3	3
**41**	3	3	3	3	3	3	3	3	3	3
**42**	3	3	3	3	3	3	3	3	3	3
**43**	3	3	3	3	3	3	3	3	3	3
**44**	0	0	0	0	3	3	3	3	3	3
**45**	0	0	0	0	3	3	3	3	3	3
**46**	0	0	3	3	3	3	3	3	3	3
**47**	2	2	3	3	3	3	3	3	3	3
**48**	0	0	3	3	3	3	3	3	3	3
**49**	3	3	3	3	3	3	3	3	3	3
**50**	0	0	3	3	3	3	3	3	3	3

Table S10. Scores obtained by the two radiologists for MCA detectability for 40 data. Related to Table 1.

No.	The MCA detectability									
	**4 Projections**		**6 Projections**		**8 Projections**		**10 Projections**		**12 Projections**	
	R1	R2	R1	R2	R1	R2	R1	R2	R1	R2
**11**	0	0	3	3	3	3	3	3	3	3
**12**	0	0	0	0	3	3	3	3	3	3
**13**	0	0	3	3	3	3	3	3	3	3
**14**	3	3	3	3	3	3	3	3	3	3
**15**	3	3	3	3	3	3	3	3	3	3
**16**	3	3	3	3	3	3	3	3	3	3
**17**	3	3	3	3	3	3	3	3	3	3
**18**	0	0	3	3	3	3	3	3	3	3
**19**	0	0	3	3	3	3	3	3	3	3
**20**	3	3	3	3	3	3	3	3	3	3
**21**	3	3	3	3	3	3	3	3	3	3
**22**	0	0	3	3	3	3	3	3	3	3
**23**	0	0	0	0	3	3	3	3	3	3
**24**	2	3	3	3	3	3	3	3	3	3
**25**	3	3	3	3	3	3	3	3	3	3
**26**	3	3	3	3	3	3	3	3	3	3
**27**	3	3	3	3	3	3	3	3	3	3
**28**	3	3	3	3	3	3	3	3	3	3
**29**	0	0	0	0	3	3	3	3	3	3
**30**	0	0	3	3	3	3	3	3	3	3
**31**	3	3	3	3	3	3	3	3	3	3
**32**	2	3	3	3	3	3	3	3	3	3
**33**	0	0	0	0	0	0	3	3	3	3
**34**	2	3	3	3	3	3	3	3	3	3
**35**	2	3	3	3	3	3	3	3	3	3
**36**	0	0	0	0	3	3	3	3	3	3

**37**	0	0	2	3	3	3	3	3	3	3
**38**	0	0	3	3	3	3	3	3	3	3
**39**	2	0	3	0	3	3	3	3	3	3
**40**	0	0	3	3	3	3	3	3	3	3
**41**	3	3	3	3	3	3	3	3	3	3
**42**	3	3	3	3	3	3	3	3	3	3
**43**	3	3	3	3	3	3	3	3	3	3
**44**	0	0	0	0	3	3	3	3	3	3
**45**	0	0	0	0	3	3	3	3	3	3
**46**	0	0	3	3	3	3	3	3	3	3
**47**	2	2	3	3	3	3	3	3	3	3
**48**	0	0	3	3	3	3	3	3	3	3
**49**	3	3	3	3	3	3	3	3	3	3
**50**	0	0	3	3	3	3	3	3	3	3

Table S11. Total scores obtained by the two radiologists for 40 data. Related to Table 1.

**No.**	**Total scores**									
	**4 Projections**		**6 Projections**		**8 Projections**		**10 Projections**		**12 Projections**	
	R1	R2	R1	R2	R1	R2	R1	R2	R1	R2
**11**	3	3	10	10	16	16	16	16	16	16
**12**	3	3	4	4	11	11	15	15	15	15
**13**	4	4	13	13	15	15	15	15	16	16
**14**	9	9	13	13	16	16	16	16	16	16
**15**	12	12	14	14	15	15	16	16	16	16
**16**	10	10	13	13	15	15	15	15	16	16
**17**	13	13	14	14	16	16	16	16	16	16
**18**	4	4	10	10	15	15	16	16	16	16
**19**	4	4	13	13	14	14	16	15	16	16
**20**	13	13	15	15	16	16	16	16	16	16
**21**	10	12	12	14	13	15	15	15	16	16
**22**	2	4	13	15	15	15	16	16	16	16
**23**	4	4	4	5	14	15	16	15	16	16
**24**	12	13	13	15	15	15	15	16	16	16
**25**	13	13	14	15	16	16	16	16	16	16
**26**	11	13	13	15	15	16	16	16	16	16
**27**	13	13	14	14	16	16	16	16	16	16
**28**	10	11	12	15	14	15	15	16	16	16
**29**	0	0	2	3	14	15	15	15	16	16
**30**	1	4	11	14	13	15	15	16	16	16
**31**	10	13	13	15	14	16	16	16	16	16
**32**	13	14	15	15	16	16	16	16	16	16
**33**	0	0	1	1	2	2	11	11	13	13
**34**	12	13	14	15	15	15	15	16	16	16
**35**	9	10	13	15	16	16	16	16	16	16
**36**	0	0	0	0	15	15	16	16	16	16

**37**	3	4	12	13	15	15	16	16	16	16
**38**	0	0	10	10	13	13	14	14	15	15
**39**	12	11	14	11	16	14	16	16	16	16
**40**	1	1	13	13	15	15	15	15	16	16
**41**	11	13	15	15	16	16	16	16	16	16
**42**	11	13	14	15	11	16	16	16	16	16
**43**	13	13	13	15	16	16	16	16	16	16
**44**	0	0	3	3	15	15	16	16	16	16
**45**	0	0	0	0	15	15	16	16	16	16
**46**	0	4	13	15	15	16	16	16	16	16
**47**	12	11	16	16	16	16	16	16	16	16
**48**	3	4	12	15	15	15	15	16	16	16
**49**	14	14	14	15	16	16	16	16	16	16
**50**	3	4	13	14	16	15	16	16	16	16

Table S12. (In Supplementary Excel) Scores obtained by two radiologists to analyze the overall image performance and vessel detectability using the 8 projection-method from external centers. Related to Table 2.

Table S13. (In Supplementary Excel) Diagnosis confidences obtained by two radiologists for reconstructed images from multicenter. Related to Table 3.

Table S14. Comparison of objective and subjective evaluation results obtained at different aneurysm stages. Data are presented as mean ± SD. Related to STAR METHODS.

	Tiny (＜3mm, n=21)	Small (3~7mm, n=37)	Others (n=24)	p value [a]
**Objective evaluation**				
**MAE**	.001104±.000450	.001050±.000446	.000894±.000259	0.16
**MSE**	.000136±.000069	.000124±.000057	.000114±.000062	0.46
**PSNR**	39.24±2.35	39.50±2.08	39.88±2.01	0.46
**SSIM**	.984±.011	.9849±.0135	.9895±.0043	0.19
**Subjective evaluation**				
**Reader 1**	4.17±0.89	4.20±0.90	4.33±0.92	0.70
**Reader 2**	4.17±0.78	4.09±0.89	4.25±0.90	0.71
**κ**	0.87	0.93	0.95	/
**p value [b]**	<0.001	<0.001	<0.001	/

a). The Kruskal–Wallis test was conducted to compare the objective indexes and the diagnostic confidence recorded by the same observer during the diagnosis of different aneurysm stages.

b). The quadratic weighted κ test was conducted to analyze the consistency of the diagnostic confidence of different observers at different stages of aneurysms.
