# Supplementary material for: Self-supervised learning enables 3D digital subtraction angiography reconstruction from ultra-sparse 2D projection views: A multicenter study
Source: Cell Rep Med. 2022 Oct 7;3(10):100775. doi: 10.1016/j.xcrm.2022.100775 (PMC9589028; doi:10.1016/j.xcrm.2022.100775)
Supplement: Document S2. Article plus supplemental information [file mmc6.pdf]

Cell Reports Medicine

Article

Self-supervised learning enables 3D digital subtraction angiography reconstruction from ultra-sparse 2D projection views: A multicenter study

Graphical abstract

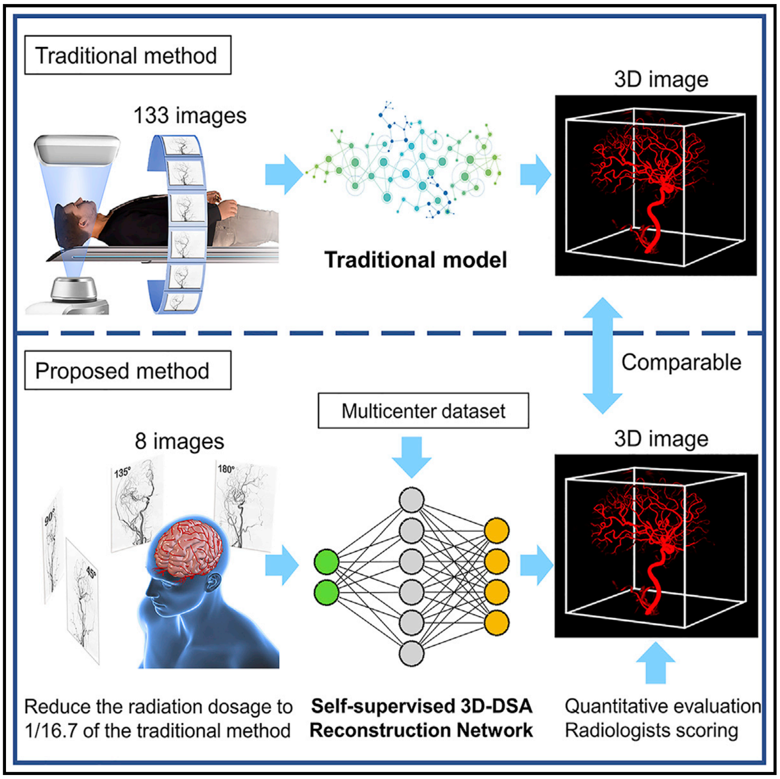

Highlights

- 3D-DSA reconstruction is established using a self-supervised learning model
- The number of 2D projections required for the model is far less than for clinical use
- Two radiologists score the reconstructed images and identify all 82 lesions

Authors

Huangxuan Zhao, Zhenghong Zhou, Feihong Wu, ..., Fan Yang, Xinggang Wang, Chuansheng Zheng

Correspondence

18942931650@163.com (J.M.), fyang@hust.edu.cn (F.Y.), xgwang@hust.edu.cn (X.W.), hqzcsxh@sina.com (C.Z.)

In brief

Zhao et al. develop a self-supervised learning method to realize 3D digital subtraction angiography (3D-DSA) reconstruction using ultra-sparse 2D projections. High-quality 3D-DSA images of 202 patients from three hospitals are reconstructed with high quality. This method may facilitate the development of a revolutionary 3D-DSA reconstruction method for use in clinic.

Zhao et al., 2022, Cell Reports Medicine *3*, 100775
October 18, 2022 © 2022
https://doi.org/10.1016/j.xcrm.2022.100775

CellPress

Cell Reports Medicine

CellPress
OPEN ACCESS

Article

Self-supervised learning enables 3D digital subtraction angiography reconstruction from ultra-sparse 2D projection views: A multicenter study

Huangxuan Zhao,[1,2,7] Zhenghong Zhou,[3,7] Feihong Wu,[1,2,7] Dongqiao Xiang,[1,2,7] Hui Zhao,[4] Wei Zhang,[1,2] Lin Li,[1,2] Zhong Li,[4] Jia Huang,[1,2] Hongyao Hu,[4] Chengbo Liu,[5] Tao Wang,[6] Wenyu Liu,[3] Jinqiang Ma,[1,2,*] Fan Yang,[1,2,*] Xinggang Wang,[3,*] and Chuansheng Zheng[1,2,8,*]

[1]Department of Radiology, Union Hospital, Tongji Medical College, Huazhong University of Science and Technology, Wuhan 430022, China
[2]Hubei Province Key Laboratory of Molecular Imaging, Wuhan 430022, China
[3]School of Electronic Information and Communications, Huazhong University of Science and Technology, Wuhan 430074, China
[4]Department of Interventional Radiology, Renmin Hospital of Wuhan University, Wuhan 430060, China
[5]Research Laboratory for Biomedical Optics and Molecular Imaging, Shenzhen Institutes of Advanced Technology, Chinese Academy of Sciences, Shenzhen 518055, China
[6]Department of Respiratory and Critical Care Medicine, University of Chinese Academy of Sciences Shenzhen Hospital, Shenzhen 518107, China
[7]These authors contributed equally
[8]Lead contact
*Correspondence: 18942931650@163.com (J.M.), fyang@hust.edu.cn (F.Y.), xgwang@hust.edu.cn (X.W.), hqzcsxh@sina.com (C.Z.)
https://doi.org/10.1016/j.xcrm.2022.100775

SUMMARY

3D digital subtraction angiography (DSA) reconstruction from rotational 2D projection X-ray angiography is an important basis for diagnosis and treatment of intracranial aneurysms (IAs). The gold standard requires approximately 133 different projection views for 3D reconstruction. A method to significantly reduce the radiation dosage while ensuring the reconstruction quality is yet to be developed. We propose a self-supervised learning method to realize 3D-DSA reconstruction using ultra-sparse 2D projections. 202 cases (100 from one hospital for training and testing, 102 from two other hospitals for external validation) suspected to be suffering from IAs were conducted to analyze the reconstructed images. Two radiologists scored the reconstructed images from internal and external datasets using eight projections and identified all 82 lesions with high diagnostic confidence. The radiation dosages are approximately 1/16.7 compared with the gold standard method. Our proposed method can help develop a revolutionary 3D-DSA reconstruction method for use in clinic.

INTRODUCTION

Subarachnoid hemorrhage is a severe subtype of stroke associated with permanent brain damage or death. The average age of patients suffering from subarachnoid hemorrhage is 55 years. In approximately 85% of the cases, subarachnoid hemorrhage is caused by intracranial aneurysms (IAs). The mortality rate for aneurysmal subarachnoid hemorrhage is approximately 50%, and 10%–15% of the patients die at home or during transportation to a hospital.[1,2] Although the rate of survival recorded for patients suffering from subarachnoid hemorrhage has increased by 17% over the past few decades, long-term cognitive impairments accompanying subarachnoid hemorrhage affect the daily work and quality of life of the patients.[3] Two-dimensional (2D) and 3D images recorded using the digital subtraction angiography (DSA) technique can be analyzed to directly visualize the bleeding points. This method has long been used as the gold standard for the diagnosis of IAs.[4,5] DSA is also an indispensable technology that is used for interventional therapy. It has been reported[5–7] that high-definition and intricate 3D images can be reconstructed using the rotational 2D projection X-ray angiography technique, which is superior to the 2D planar imaging technique. The most classic 3D-DSA reconstruction method involves the use of the Feldkamp-Davis-Kress (FDK) algorithm.[8] The steps followed to obtain the 3D-DSA reconstruction data have been reported: a patient lies on the operating table for DSA, and the brain of the patient is scanned using the DSA technique. A small amount of iodixanol (contrast agent) is injected into the patient's carotid artery. Following this, the patient's brain is scanned again using the DSA technique. During the scanning process, the C-arm is rotated by ~199.5° over ~5 s keeping the position of the fulcrum unchanged (0°). During the process, ~133 2D images are recorded. The images recorded before and after the injection of the contrast agent are labeled as the mask and dynamic images, respectively. 2D-DSA images are generated by subtracting the mask image from the corresponding dynamic image. The 3D-DSA image is obtained by reconstructing the ~133 2D-DSA images using the FDK-based 3D reconstruction

This is an open access article under the CC BY-NC-ND license (http://creativecommons.org/licenses/by-nc-nd/4.0/).

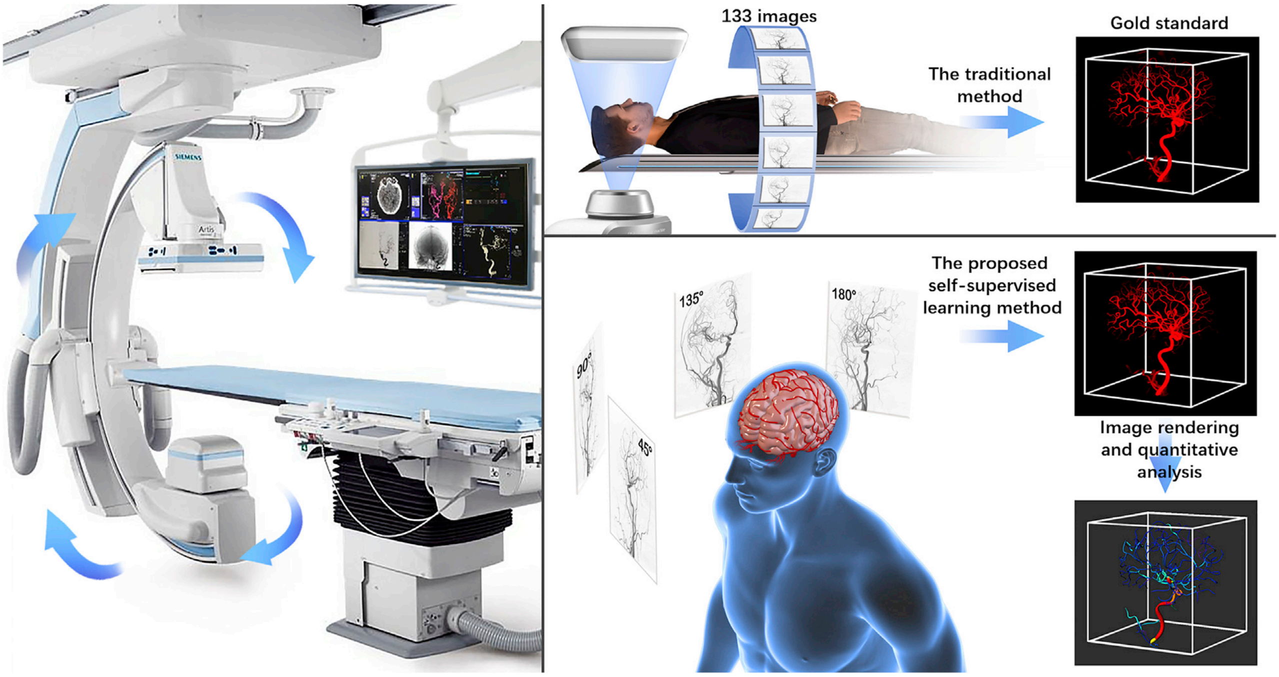

Figure 1. Schematic representation of the 3D-DSA reconstruction of samples using the gold standard method and the method proposed herein

algorithm. The FDK-based algorithm was revised and used by Siemens, General Electric, Philips, and other medical device manufacturers for years.[8–10] It has long been used as the gold standard in clinic. This traditional 3D-DSA scanning and reconstruction method (the gold standard) requires the patient to be continuously exposed to radiation. To date, effective methods that can help significantly reduce the radiation dosage (ensuring the quality of the reconstructed images) have not been reported.

Deep learning (DL) is a type of machine learning technique that uses multi-layered artificial neural networks for the automated analysis of signals or data.[11–13] Convolutional neural networks (CNN) are a popular embodiment of the DL technique that can be used for fitting nonlinear equations using 2D or 3D images as the input data. The technique does not require the use of manually designed image features. Some CNN-based image reconstruction methods based on sparse sampling data have been developed and used to record images using magnetic resonance imaging, photoacoustic imaging, and other medical/biomedical imaging techniques.[14–19] Most of the developed methods are based on image super-resolution and enhancement algorithms used in the field of computer vision. Recent years have seen the development of a few 2D (X-ray)-3D computed tomography (CT) reconstruction methods that can be used in the field of medical imaging: (1) a patient-specific 2D-3D reconstruction method[20] is used to obtain the 3D data of each patient for personalized training. In this case, 2D data need not be used to reconstruct the 3D images if the 3D-DSA technique is used to record the relevant images. (2) Two 2D-3D reconstruction methods based on two projection views have also been reported.[21,22] These methods exhibit good reconstruction effects when samples (such as knee bone and coronary artery) for which detailed data are absent are studied. The methods are not suitable to study cases where detailed information on vasculature needs to be reconstructed.

Herein, we report the development of a self-supervised 3D-DSA reconstruction (SSDR) network to reconstruct 3D-DSA images from ultra-sparse 2D projection views. While the gold standard requires the analysis of ∼133 2D images, our method can be used to efficiently reconstruct multi-scale human cerebral vasculature characterized by the presence of detailed microvessels from eight 2D images (Figure 1). Thus, the patient is subjected to 1/16.7th of the radiation dosage used during the gold standard imaging method. The 3D reconstruction process does not require annotation by radiologists, and 3D data are not used for supervision and training while using the SSDR method. Instead, we developed a novel self-supervised learning method to train the 3D reconstruction network using sparse 2D views. Once the model is trained, it can be used to reconstruct 3D images in real time.

A total of 202 DSA cerebrovascular imaging datasets from patients suspected to be suffering from cerebral aneurysms were retrieved from the imaging databases of three hospitals: Wuhan Union Hospital (institution I), Wuhan Union West Hospital (institution II), and Hubei Provincial People's Hospital (institution III). Among these, a total of 100 cases were retrieved from the Wuhan Union Hospital in the period ranging from December 2020 to March 2021; 50 cases were used for training, and the other 50 cases were used for testing. Fifty-four cases were retrieved from the Wuhan Union West Hospital in the period ranging from January to February 2022, and 48 cases were retrieved from the Hubei Provincial People's Hospital in the period ranging from January to February 2022. The data from

CellPress
OPEN ACCESS

the two centers were used for external validation. The flowchart corresponding to the process of data acquisition and division is shown in Figure S1. We realized the 3D-DSA reconstruction of the data, performed the image rendering and quantitative analyses, and used different colors to identify blood vessels of different diameters to quickly distinguish between and identify lesions. The details have been presented in the STAR Methods and results sections. The SSDR network is expected to revolutionize the 3D-DSA reconstruction technology used in clinic.

RESULTS

Overall framework and performance of the SSDR network

The DL method was developed with the aim of using it in clinic. The framework of the SSDR network is presented in Figure 2A. Multiple (4, 6, 8, 10, and 12) 2D projections from different view angles of the 3D-DSA images were used as the inputs for our network. The angles corresponding to the 4, 6, 8, 10, and 12 projection views are listed in Table S1. The used angles covered 180°. We experimentally demonstrated that the reconstruction results are affected when limited angle projections are used as input, as shown in the reconstruction results with limited angle projections of the STAR Methods and Table S2. The self-supervised 3D-DSA reconstruction process proceeds over three modules: the multi-view preprocessing module, the reconstruction network, and the self-supervised module. Using the multi-view preprocessing module, we analyzed the 2D and 3D feature representations following resizing, angle-based dimensional ascending, and convolution processes. Herein, we used the key step of the backpropagation (BP) algorithm:[23,24] each input 2D image was back-projected according to the corresponding viewing angle to obtain the 3D data. These 3D data were further input to the next module as different channels. In the reconstruction module, 3D reconstruction is realized using a 3D U-Net[25] system that contains the details of encoding and decoding operations. In the self-supervised module, the 2D-DSA images are obtained from the reconstructed 3D-DSA images by analyzing the differentiable projections and using a loss function to determine the difference between the input image and the projected 2D-DSA image (recorded at the same angle). The training process followed by us proceeds over two stages: the first stage uses low-resolution images for 3D-DSA reconstruction, and its prediction result is fused with the high-resolution images to predict the high-resolution 3D-DSA image. A detailed description of these two stages is presented in the detailed description of the two stages of the SSDR network of the STAR Methods and Figure S2. During the process of testing, we directly input the multi-view 2D images into the DL models to rapidly obtain the corresponding 3D reconstruction results.

To verify whether 50 cases were enough for training, we used 75 and 100 cases isolated from institution I to train models under conditions of the same iteration numbers (400 epochs) and tested these three models using the 102 cases obtained from the other two centers. The peak signal-to-noise ratio (PSNR), structural similarity index (SSIM), mean square error (MSE), and mean absolute error (MAE) of the reconstruction results obtained using the eight projections were calculated (Table S3). We observed that when the number of cases used for training was increased to 100, the quantitative results remained unaffected. This proves that unless the training data were increased in multiple-rate, little effect was exerted on the results.

The reconstruction ability of the SSDR network was studied. Figure 2B presents the anteroposterior and lateral views of the 3D reconstructed images of a patient suffering from an aneurysm. 4, 6, 8, 10, and 12 2D projections were used as the inputs. The rendering results obtained by analyzing the 3D reconstructed images presented in Figure 2B are shown in Video S1. There are two reasons for choosing these two projections for comparing the groups: (1) lateral (90°) and anteroposterior (180°) projections are used as supervised projections for every group (for all 4, 6, 8, 10, and 12 projections). The reconstruction quality obtained can intuitively reflect the quality of the trained models. (2) Lateral and anteroposterior positions are standard medical positions. Aneurism can be clearly observed in Figure 2B, and it is indicated with yellow arrows in these two figures. The 3D reconstructed images are comparable to the images recorded using the gold standard method. It is difficult to differentiate the images with naked eyes. We further quantified and determined the efficiency of the method by analyzing the loss curves (Figures 2C–2H). Figure 2C presents a summary of all the loss curves. Steel-blue, goldenrod, tomato-red, cyan, and slate-gray lines represent the training loss curves of the reconstructed results obtained using 4, 6, 8, 10, and 12 projections, respectively. The enlarged images of the training loss curves are presented in Figures 2D–2H. Analysis of the loss curves indicates that the models are trained to fit the training data well. This was manually confirmed (Figure 2B).

Quantitative analysis

The process of 3D quantitative analysis was used to further verify the efficiency of the SSDR network (Figure 3). Figures 3A–3F present the quantitative results obtained for the vascular and lesion diameters of a randomly selected patient. The 3D reconstructed models are shown in Video S2. The quantification method used for the analysis of the vascular and lesion diameters is an intuitive and high-standard evaluation method. Subtle changes in the reconstructed pattern are intuitively reflected in the results. This method can efficiently assist radiologists in locating and identifying lesions as color mutations are observed in the areas bearing the lesion. It has been observed that it is gradually becoming easy to obtain good quantitative results from the 3D reconstruction images obtained using 4, 6, 8, 10, and 12 projections. As indicated by the red (two) and cyan (one) arrows, significant levels of differences in the color can be observed between Figures 3A and 3F. The vessels and the lesion presented in Figure 3B appear thick. The vessels and the lesion presented in Figures 3C–3E are similar to those presented in Figure 3F. The four key indicators (SSIM, PSNR, MSE, and MAE)[26] were further used to quantify the reconstruction results obtained for the internal testing data obtained from the patients (institution I). A detailed description of these four indicators can be found in the STAR Methods section. The five color bars (steel-blue, goldenrod, tomato-red, cyan, and slate-gray) presented in Figure 3G reveal the results obtained from the quantitative studies conducted using the 3D reconstruction images. The

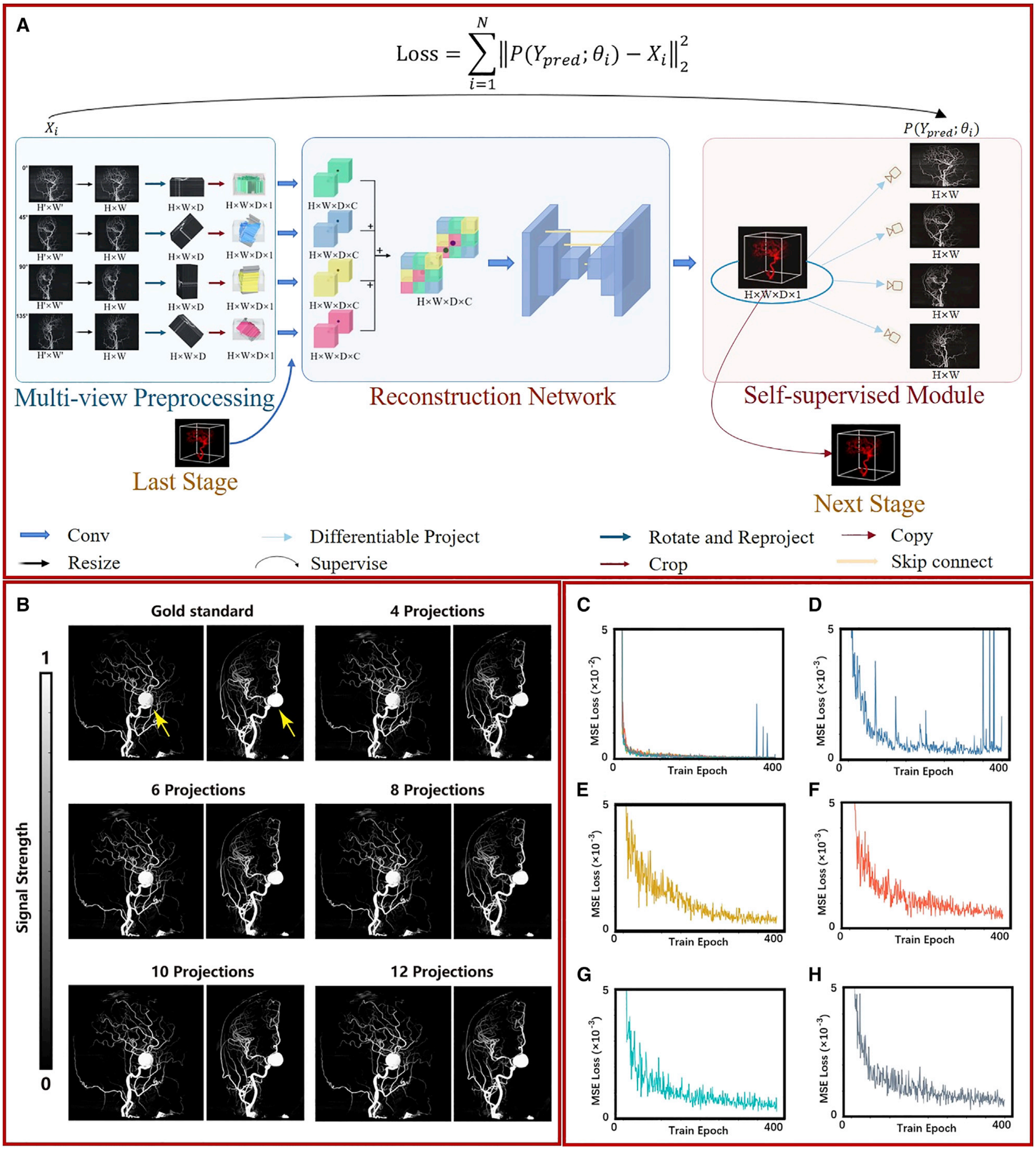

Figure 2. Overall framework and reconstruction results of the SSDR network

(A) Overall framework of the SSDR network.

(B) Lateral and anteroposterior views of the 3D reconstructed images obtained using 4, 6, 8, 10, and 12 2D projections as inputs recorded for a typical patient suffering from aneurysms.

(C–H) Training loss curves. Steel-blue, goldenrod, tomato-red, cyan, and slate-gray lines represent the training loss curves of the reconstructed results obtained using 4, 6, 8, 10, and 12 projections, respectively.

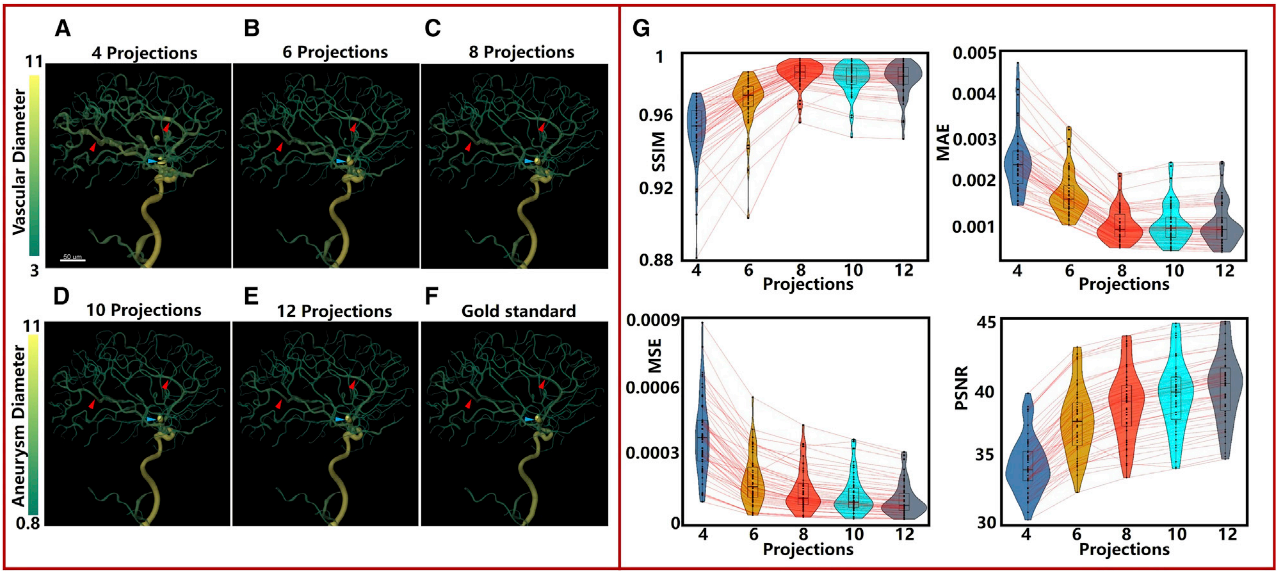

Figure 3. Results obtained using the 3D quantitative analysis method

(A–F) Quantification results obtained for the vascular and lesion diameters of a randomly selected patient. Different diameters are represented by different colors.
(G) Results from quantitative analysis displayed in violin plots for the four key indicators (SSIM, PSNR, MSE, and MAE) of internal testing patients (institution I).

colors represent the results obtained when 4, 6, 8, 10, and 12 projections were used, respectively. The values of the four indicators are presented in Table S4 as mean ± standard deviation (SD). Analysis of Figure 3G reveals that as the number of input projections increases, better PSNR and MSE values are obtained. The SSIM and MAE values basically conform to this rule. Excellent reconstruction results (corresponding to the indicators) were obtained using eight projections. The mean SSIM, PSNR, MSE, and MAE values were 0.987, 38.69, 1.58×10^{-4}, and 1.05×10^{-3}.

Evaluation of image quality and diagnostic confidence by radiologists

Currently, there are no radiologist scoring methods that can be effectively used to conduct these studies. We developed a comprehensive scoring method (Table S5) for evaluating the 3D reconstruction results based on the results presented in previously reported papers dealing with the subjective evaluation of vascular visualization.[27,28] The reconstructed image performance, vessel detectability, and the diagnostic confidence for vascular pathology were analyzed by radiologists. Two radiologists with 8 (reader 1, R1) and 7 (reader 2, R2) years of experience randomly selected 10 cases (nos. 1–10) out of the 50 tested cases from institution I. They evaluated a total of 30 images, reconstructed from four, six, and eight projections to verify the repeatability of the developed scoring method. The results (Table S6) reveal a good interobserver agreement: the κ value (quadratic weighted kappa test was used to obtain the values) of the overall image performance, blood vessel recognition for internal carotid artery (ICA), anterior cerebral artery (ACA), and middle cerebral artery (MCA), were 0.853, 0.792, 0.902, and 0.902, respectively. The results indicate that the scoring method is highly repeatable and can be efficiently used to conduct this study.

Table 1 presents the evaluation results obtained by the radiologists during the analysis of the 3D images of the remaining 40 tested datasets (nos. 11–50) in institution I. The images were reconstructed from 4, 6, 8, 10, and 12 projections. The detailed scoring results for every patient have been presented in Tables S7–S11. The Friedman test was conducted to obtain the p values to compare the data obtained using 4, 6, 8, 10, and 12 projections (post-hoc pairwise comparisons). Among the five types of reconstruction results, the total score of the reconstructed images obtained using four projections was the lowest (7.28 ± 5.15). The total score of the reconstructed images obtained using six projections (11.5 ± 4.6) was significantly higher than that obtained using four projections. This is primarily reflected by the degree of improvement in visualization achieved for the sub-branches of ACA and MCA. It should be noted that the overall image performance and the degree of visualization corresponding to ICA need to be improved further. The total scores recorded for the reconstructed images obtained using 10 and 12 projections (15.59 ± 0.88 and 15.88 ± 0.52, respectively) were close 16, the maximum score. The total score of the reconstructed images obtained using eight projections (14.71 ± 2.29) was slightly lower than the total score of the reconstructed images obtained using 10 projections. A significant difference in the imaging performance or vessel detectability was not observed ($p > 0.05$). This indicates that high-quality images can be recorded using the minimum number of projections when the method that used eight projections as the input is followed.

External validation

External data from institutions II and III were used to validate the generalization ability of the proposed method. The overall performance (Figures 4A and 4B), results from quantitative analyses

Table 1. Overall image performance and vessel detectability studied by the radiologists

		4 projections	6 projections	8 projections	10 projections	12 projections	p value
The overall image performance		1.29 ± 1.53[a,b,c]	2.63 ± 1.62[d,e,f]	4.1 ± 1.15[g]	4.64 ± 0.74	4.9 ± 0.38	<0.001
Vessel detectability	ICA	2.99 ± 1.43[h,a,b,c]	3.98 ± 1.17[d,e,f]	4.76 ± 0.54	4.95 ± 0.22	4.98 ± 0.16	<0.001
	ACA	1.55 ± 1.5[a,b,c]	2.48 ± 1.15	2.93 ± 0.47	3 ± 0	3 ± 0	<0.001
	MCA	1.45 ± 1.44[h,a,b,c]	2.43 ± 1.16	2.93 ± 0.47	3 ± 0	3 ± 0	<0.001
Total score		7.28 ± 5.15[h,a,b,c]	11.5 ± 4.6[d,e,f]	14.71 ± 2.29[g]	15.59 ± 0.88	15.88 ± 0.52	<0.001

Data are presented as mean ± SD. Friedman test was conducted to obtain the p values to compare the data obtained using 4, 6, 8, 10, and 12 projections (post-hoc pairwise comparisons). ICA, internal carotid artery; ACA, anterior cerebral artery; MCA, middle cerebral artery.
[a]Adjusted $p < 0.05$, comparison between scores of 4 and 8 projections by post-hoc pairwise comparisons.
[b]Adjusted $p < 0.05$, comparison between scores of 4 and 10 projections by post-hoc pairwise comparisons.
[c]Adjusted $p < 0.05$, comparison between scores of 4 and 12 projections by post-hoc pairwise comparisons.
[d]Adjusted $p < 0.05$, comparison between scores of 6 and 8 projections by post-hoc pairwise comparisons.
[e]Adjusted $p < 0.05$, comparison between scores of 6 and 10 projections by post-hoc pairwise comparisons.
[f]Adjusted $p < 0.05$, comparison between scores of 6 and 12 projections by post-hoc pairwise comparisons.
[g]Adjusted $p < 0.05$, comparison between scores of 8 and 12 projections by post-hoc pairwise comparisons.
[h]Adjusted $p < 0.05$, comparison between scores of 4 and 6 projections by post-hoc pairwise comparisons.

(Figures 4C and 4D), and subjective scores (Tables 2 and 3) were evaluated. Figures 4A and 4B present the anteroposterior and lateral views of the 3D reconstructed images of two randomly selected patients, one selected from each of the two institutions. In these cases, 4, 6, 8, 10, and 12 2D projections were used as inputs. Analysis of Figure 4A reveals that a little loss in layer information is observed when four projections are used to reconstruct images. The loss is represented by red arrows. This phenomenon is observed during the post-processing method for a few of the reconstructed images formed using four projections. The occurrence of this phenomenon can be avoided by adjusting the threshold. A detailed description of the post-processing method has been presented in the detailed description of the post-processing method of the STAR Methods and Figure S3. Analysis of Figure 4 also reveals that the external validation data are in good agreement with the internal test data. The results obtained from the quantitative analyses revealed that significant differences were not observed for the external centers for the reconstruction results obtained using the 8, 10, or 12 projections. Parameters of institutions II and III were slightly better than those of institution I. The mean SSIM, PSNR, MSE, and MAE values recorded for the reconstruction results obtained using the eight projections were 0.989, 40.06, 1.06×10^{-4}, and 8.6×10^{-4} (Figure 4C) and 0.981, 39.58, 1.32×10^{-4}, and 1.23×10^{-3} (Figure 4D) for institutions II and III, respectively, and the mean SSIM, PSNR, MSE, and MAE values were 0.987, 38.69, 1.58×10^{-4}, and 1.05×10^{-3} (Figure 3G) for institution I. The values of these four indicators are presented in detail in Table S4. The values are presented as mean ± SD.

Table 2 presents the results obtained by the radiologists during the analysis of the multicenter 3D images (nos. 11–50 of institution I, nos. 1–54 of institution II, and nos. 1–48 of institution III) obtained using eight projections. The detailed scoring results for every patient admitted to institutions II and III have been presented in Table S12. The Kruskal-Wallis test was conducted to obtain the p values to compare the data obtained using the multicenter eight projections method. The data presented in the table reveal that the evaluation results obtained for the patients admitted to institution III were slightly better than those obtained for the patients admitted to the other two centers. Significant differences were not observed between the four indicators (overall image performance, ICA, ACA, and MCA) corresponding to the three centers.

We studied whether the reconstructed 3D images obtained from eight projections could meet the requirements of the radiologists for diagnosis. Table 3 presents the diagnosis confidence (for the two radiologists) obtained during the identification of the lesions present in these reconstructed multicenter images obtained using eight projections. The detailed scoring method has been presented in the STAR Methods section, and the detailed scoring results for every patient are shown in Table S13. High diagnosis confidence was obtained by R1 and R2 during the diagnosis of the pathological and non-pathological vessels. All scoring results were greater than 4 points (4 points = basically certain). The radiologists correctly identified all 82 aneurysms (27, 26, and 29 aneurysms were identified from institutions I, II, and III, respectively) from the 3D reconstructed images without knowing the results beforehand. In addition, the diagnostic confidence reached good interobserver consistency. The κ values corresponding to the diagnostic confidence were 0.93, 0.91, and 0.90 for pathological vessels associated with institutions I, II, and III, respectively, and 0.75, 0.90, and 0.87 for non-pathological vessels associated with institutions I, II, and III, respectively.

We classified 82 lesions for more detailed quantification, and the classified method is described in the subjective and objective evaluations of the STAR Methods section. Among the 82 lesions, 21 were divided into the tiny aneurysm group, 37 were divided into the small aneurysm group, and the remaining 24 were divided into the "other" group. We scored the reconstruction results obtained at different groups using subjective and objective evaluation methods, and the scoring results are shown in Table S14. Two cases from each group were randomly selected for presentation, as shown in Figure S4. The Kruskal-Wallis test was used to compare the differences between the objective indices and the

CellPress
OPEN ACCESS

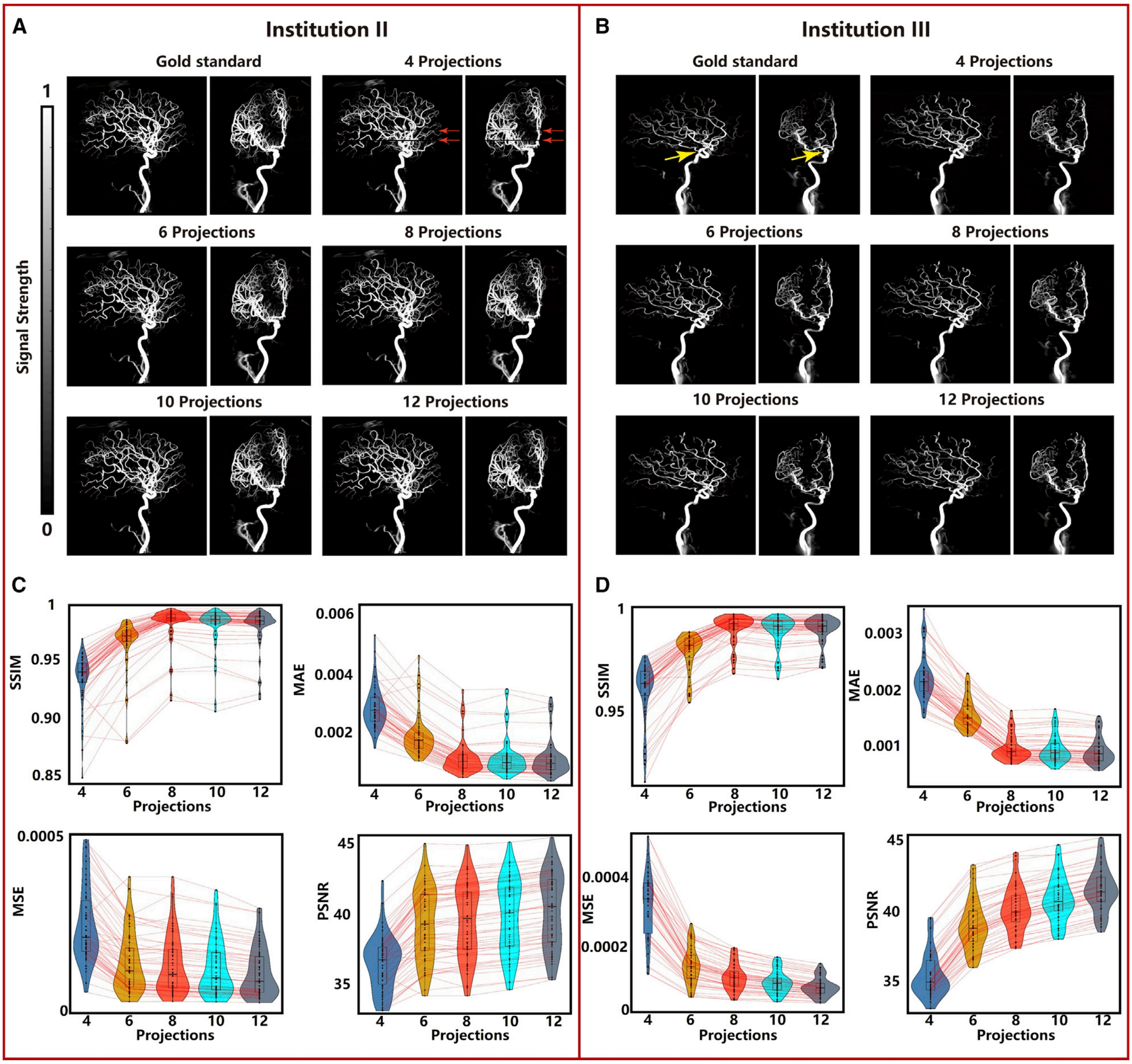

Figure 4. External validation results

(A and B) Anteroposterior and lateral views of the 3D reconstructed images recorded using 4, 6, 8, 10, and 12 2D projections as inputs for two randomly selected patients, one from each of the two institutions.

(C–D) Results from quantitative analysis displayed in the violin plots corresponding to the four key indicators (SSIM, PSNR, MSE, and MAE). Values corresponding to the (C) 54 patients admitted to institution II and (D) 48 patients admitted to institution III.

diagnostic confidence during the diagnosis of different groups. The quadratic weighted κ test was used to analyze the consistency of the diagnostic confidence of different observers. The data are presented as mean ± SD. For the case of the objective indicators, the values of the four indicators (SSIM, MAE, MSE, and PSNR) improved by a small degree with the progression of the lesion stages. Statistical differences between the groups (the p values were 0.16, 0.46, 0.46, and 0.19, respectively) were not observed (Table S14). For the case of the subjective indicators, the scores corresponding to the observer's diagnostic confidence recorded at different stages of aneurysms were all >4 points, and statistical difference between the groups was not observed (the p values were 0.70 and 0.71, respectively). High diagnostic confidence and interobserver agreement were realized for R1 and R2 at different lesion stages. The interobserver agreement increases (for tiny, small, and "others" group, the κ values are 0.87, 0.93, and 0.95, respectively) with the progression of the lesion stage. The results revealed that the reconstruction results obtained for the tiny and small aneurysms could be effectively used for disease diagnosis.

Table 2. Comparison between the overall image performance and vessel detectability

Institution		I	II	III	p value
The overall image performance		4.1 ± 1.15	4.31 ± 0.54	4.45 ± 0.53	0.340
Vessel detectability	ICA	4.76 ± 0.54	4.81 ± 0.46	4.88 ± 0.43	0.144
	ACA	2.93 ± 0.47	2.94 ± 0.31	2.99 ± 0.07	0.589
	MCA	2.93 ± 0.47	2.87 ± 0.39	2.96 ± 0.14	0.203
Total score		14.71 ± 2.29	14.92 ± 1.19	15.27 ± 0.84	0.183

The data were obtained using the multicenter eight-projection method. Data are presented as mean ± SD. Kruskal-Wallis test was conducted to obtain the p values that compare the data obtained using the multicenter eight-projection method. ICA, internal carotid artery; ACA, anterior cerebral artery; MCA, middle cerebral artery.

Table 3. Diagnostic confidence corresponding to vascular pathology

	Reader 1	Reader 2	κ	p value
Institution I				
Pathological vessel (n = 27)	4.21 ± 1.08	4.10 ± 0.99	0.93	<0.001
Non-pathological vessel (n = 13)	4.26 ± 1.08	4.16 ± 1.00	0.75	0.005
Institution II				
Pathological vessel (n = 26)	4.19 ± 0.85	4.08 ± 0.80	0.91	<0.001
Non-pathological vessel (n = 28)	4.36 ± 0.62	4.36 ± 0.62	0.90	<0.001
Institution III				
Pathological vessel (n = 29)	4.24 ± 0.74	4.17 ± 0.73	0.90	<0.001
Non-pathological vessel (n = 19)	4.26 ± 0.65	4.26 ± 0.65	0.87	<0.001

Data are presented as mean ± SD. The interobserver reliability was observed during the diagnostic confidence evaluation process (recorded by both the readers, as measured from the quadratic weighted kappa [κ]).

DISCUSSION

Herein, we proposed a self-supervised learning method to realize 3D cerebrovascular DSA reconstruction using ultra-sparse 2D projection views. The advantages of using this self-supervised learning method have been presented. First, unlike the gold standard method, where ~133 2D-DSA images are required to obtain good reconstruction results, in this case, good reconstruction results can be achieved using 1/16.7th of the 2D images used in the gold standard method. Second, this method does not require the use of a 3D label. This is advantageous as it is difficult to obtain real 3D labels (as described in the STAR Methods section). Third, it alleviates the problem of excessive background noise in the case of 3D supervision because more than 95% of the voxels in the 3D labels are background voxels. The background ratio recorded for the 2D labels post projection is approximately 70%. This helps realize efficient supervision. 4, 6, 8, 10, and 12 2D projections were used for the studies. Excellent image quality was obtained, even if compared with the gold standard (reconstructed with ~133 2D projections). The use of the SSDR network can potentially help significantly reduce the radiation dosage received by the human body during the process of imaging. The method can be used to develop innovative technologies for DSA reconstruction.

Three methods[20–22] have been reported in the literature to realize 2D-3D medical imaging. However, these methods could not be effectively used for our studies. The SSDR network presents several advantages: (1) the multi-view preprocessing module to provide high-quality inputs for the next two stages; this module combines the key parts of the BP algorithm[23,24] with that of Kasten et.al.[21] Kasten et.al[21] only inputs different projections through multiple channels, whereas we back-project each input 2D projection according to the corresponding viewing angle to obtain the 3D data. This allows the 3D data to have a rough outline from the beginning. (2) Multi-scale features can be extracted using two cascaded networks (i.e., the low- and high-resolution reconstruction stages). (3) A novel self-supervised method is proposed, and the input 2D images are used for self-supervised training. Hence, the 3D reconstruction process does not require annotations from radiologists nor the 3D data obtained by the gold standard method for supervision. The results reveal that the SSDR network helps realize superior 3D cerebrovascular DSA reconstruction from ultra-sparse 2D projections.

Objective and subjective quantitative evaluation methods were used to evaluate the reconstruction results obtained using the SSDR network. These two evaluation methods have their advantages. SSIM, PSNR, MSE, and MAE values are used as the objective evaluation parameters. An objective evaluation can be conducted under these conditions. Under these conditions, the introduction of artificial subjective factors can be avoided. The disadvantage of the method lies in the fact that when minutely detailed areas are poorly reconstructed, the values of the quantitative parameters remain almost unaffected. Radiologists can readily identify the differences in results obtained during analytical studies. However, subjective differences are unavoidable. The results obtained using subjective and objective evaluation methods were analyzed. An increase in the input projection numbers resulted in the improvement of the overall image performance and detectability of ICA, ACA, and MCA of the reconstructed images. The noise decreased significantly, and the microvasculature gradually became clear. The diagnostic confidence recorded by the two radiologists for non-pathological vessels was higher than the diagnostic confidence recorded for the pathological vessels. This can be attributed to the fact that the course and shape of healthy cerebral vasculature are regular. Thus, accurate diagnosis is made by the radiologists. It has been observed that pathological vessels do not present a regular appearance. Even then, radiologists can identify tiny aneurysms in reconstructed images from eight projections under these conditions. We concluded that the method used to obtain the 3D reconstruction results obtained using four or six projections should be improved further. Significant statistical differences between the reconstruction results obtained using eight and 10 projections were not observed. It was observed that the performances of the 10- and 12- projection-based

methods could be improved (the performance improved with an increase in the number of projections). However, the radiation dosage used and the computational complexity of the models were significantly high. Therefore, the method based on eight projections was the most cost-effective method that could be used in clinic. The SSDR method can be executed using eight 2D projections (from different views) for 3D reconstruction, and the radiation dosage is only 1/16.7th of the dosage currently used in clinic. The currently used method uses 133 2D projections for reconstruction.

Limitations of the study

There are certain limitations to the method, and there is scope for improvement: (1) the SSDR network was built in a data-driven manner, and it may not have enough generalization ability to perfectly analyze thousands of data centers. Thus, massive validations using more data are still required before it is used in clinical applications. (2) We empirically observed that the performance of SSDR deteriorated when the borders of the input vessels could not be distinctly identified or images contained significantly high levels of noise. Thus, a denoising module should be added to the algorithm in the near future to improve the method and render it applicable for the diagnosis of more diseases. (3) Slight differences in the signal strength and background noises in the reconstructed images obtained from different centers were observed during subjective evaluation. The differences can be attributed to the differences in the input images collected using different scanner makers. However, the method does not influence the subjective and objective scoring results. This proves that effective generalization can be achieved for the images collected using these three commonly used scanner makers using SSDR. The results will be further validated using more scanner makers in the near future. (4) As the injection dosage of the contrast agents used under clinical settings is strictly regulated,[29,30] we did not discuss the generalization ability of SSDR using different contrast agents. This will be further verified in the near future by conducting animal experiments.

STAR★METHODS

Detailed methods are provided in the online version of this paper and include the following:

- KEY RESOURCES TABLE
- RESOURCE AVAILABILITY
  - Lead contact
  - Materials availability
  - Data and code availability
- EXPERIMENTAL MODEL AND SUBJECT DETAILS
- METHOD DETAILS
  - Overall description of the scientific research problem
  - Study design
  - Detailed steps of data acquisition
  - Problem description and response strategy
  - Detailed description of the two stages of the SSDR network
  - Training details
  - Detailed description of the post-processing method
- QUANTIFICATION AND STATISTICAL ANALYSIS
  - Subjective and objective evaluations
  - Evaluation of image quality and diagnostic confidence by radiologists
  - Reconstruction results with limited angle projections
  - Experiments to compare the data obtained by three different methods

SUPPLEMENTAL INFORMATION

Supplemental information can be found online at https://doi.org/10.1016/j.xcrm.2022.100775.

ACKNOWLEDGMENTS

Huangxuan Zhao, Zhenghong Zhou, Feihong Wu, and Dongqiao Xiang contributed equally to this work. We would like to thank Yun Feng from the Center for Biological Imaging (CBI), Institute of Biophysics, Chinese Academy of Science, for her help in analyzing the DSA images. We are grateful for the financial assistance provided by the National Natural Science Foundation of China (NSFC; grant no. 82102154), the Key Research and Development Projects of Hubei Province (grant no. 2020BAB022), and the China Postdoctoral Science Foundation (grant nos. 2021M691156 and 2021T140238).

AUTHOR CONTRIBUTIONS

Conception and design, F.Y., X.G.W., and C.S.Z.; methodology, H.X.Z., Z.H.Z., X.G.W., F.H.W., D.Q.X., H.Z., W.Z., and L.L.; investigation, H.X.Z., F.H.W., D.Q.X., and H.Y.H.; visualization, H.X.Z., Z.H.Z., J.Q.M., J.H., and Z.L.; obtained funding, H.X.Z. and C.S.Z.; project administration, H.X.Z., C.S.Z., and F.Y.; supervision, F.Y., X.G.W., and C.S.Z.; writing – original draft, H.X.Z., Z.H.Z., and F.H.W.; writing – review & editing, H.X.Z., X.G.W., C.S.Z., C.B.L., and W.Y.L. All authors approved the final manuscript.

DECLARATION OF INTERESTS

The authors declare no competing interests.

Received: February 14, 2022
Revised: August 4, 2022
Accepted: September 17, 2022
Published: October 7, 2022

REFERENCES

1. van Gijn, J., Kerr, R.S., and Rinkel, G.J. (2007). Subarachnoid haemorrhage. Lancet *369*, 306–318.
2. Lawton, M.T., and Vates, G.E. (2017). Subarachnoid hemorrhage. N. Engl. J. Med. *377*, 257–266.
3. Macdonald, R.L., and Schweizer, T.A. (2017). Spontaneous subarachnoid haemorrhage. Lancet *389*, 655–666.
4. van Asch, C.J.J., Velthuis, B.K., Rinkel, G.J.E., Algra, A., de Kort, G.A.P., Witkamp, T.D., de Ridder, J.C.M., van Nieuwenhuizen, K.M., de Leeuw, F.E., Schonewille, W.J., et al. (2015). Diagnostic yield and accuracy of CT angiography, MR angiography, and digital subtraction angiography for detection of macrovascular causes of intracerebral haemorrhage: prospective, multicentre cohort study. BMJ *351*, h5762.
5. Thompson, B.G., Brown, R.D., Amin-Hanjani, S., Broderick, J.P., Cockroft, K.M., Connolly, E.S., Duckwiler, G.R., Harris, C.C., Howard, V.J., Johnston, S.C.C., et al. (2015). Guidelines for the management of patients with unruptured intracranial aneurysms. Stroke *46*, 2368–2400.
6. Connolly, E.S., Rabinstein, A.A., Carhuapoma, J.R., Derdeyn, C.P., Dion, J., Higashida, R.T., Hoh, B.L., Kirkness, C.J., Naidech, A.M., Ogilvy,

C.S., et al. (2012). Guidelines for the management of aneurysmal subarachnoid hemorrhage. Stroke *43*, 1711–1737.

7. Cieściński, J., Serafin, Z., Strześniewski, P., Lasek, W., and Beuth, W. (2012). DSA volumetric 3D reconstructions of intracranial aneurysms: a pictorial essay. Pol. J. Radiol. *77*, 47–53.
8. Feldkamp, L.A., Davis, L.C., and Kress, J.W. (1984). Practical cone-beam algorithm. J. Opt. Soc. Am. A *1*, 612–619.
9. Xue, L., Suzuki, H., Ohtake, Y., Fujimoto, H., Abe, M., Sato, O., and Takatsuji, T. (2015). Numerical analysis of the feldkamp–davis–kress effect on industrial X-ray computed tomography for dimensional metrology. J. Comput. Inf. Sci. Eng. *15*.
10. Gailloud, P., Oishi, S., and Murphy, K. (2005). Three-dimensional fusion digital subtraction angiography: new reconstruction algorithm for simultaneous three-dimensional rendering of osseous and vascular information obtained during rotational angiography. AJNR. Am. J. Neuroradiol. *26*, 908–911.
11. Schmidhuber, J. (2015). Deep learning in neural networks: an overview. Neural Netw. *61*, 85–117.
12. Eslami, S.M.A., Jimenez Rezende, D., Besse, F., Viola, F., Morcos, A.S., Garnelo, M., Ruderman, A., Rusu, A.A., Danihelka, I., Gregor, K., et al. (2018). Neural scene representation and rendering. Science *360*, 1204–1210.
13. LeCun, Y., Bengio, Y., and Hinton, G. (2015). Deep learning. Nature *521*, 436–444.
14. Masutani, E.M., Bahrami, N., and Hsiao, A. (2020). Deep learning single-frame and multiframe super-resolution for cardiac MRI. Radiology *295*, 552–561.
15. Zhao, H., Ke, Z., Yang, F., Li, K., Chen, N., Song, L., Zheng, C., Liang, D., and Liu, C. (2021). Deep learning enables superior photoacoustic imaging at ultralow laser dosages. Adv. Sci. *8*, 2003097.
16. Du, J., He, Z., Wang, L., Gholipour, A., Zhou, Z., Chen, D., and Jia, Y. (2020). Super-resolution reconstruction of single anisotropic 3D MR images using residual convolutional neural network. Neurocomputing *392*, 209–220.
17. Helmerich, D.A., Beliu, G., Matikonda, S.S., Schnermann, M.J., and Sauer, M. (2021). Photoblueing of organic dyes can cause artifacts in super-resolution microscopy. Nat. Methods *18*, 253–257.
18. Davoudi, N., Deán-Ben, X.L., and Razansky, D. (2019). Deep learning optoacoustic tomography with sparse data. Nat. Mach. Intell. *1*, 453–460.
19. Zhou, S.K., Greenspan, H., Davatzikos, C., Duncan, J.S., Van Ginneken, B., Madabhushi, A., Prince, J.L., Rueckert, D., and Summers, R.M. (2021). A review of deep learning in medical imaging: imaging traits, technology trends, case studies with progress highlights, and future promises. Proc. IEEE *109*, 820–838.
20. Shen, L., Zhao, W., and Xing, L. (2019). Patient-specific reconstruction of volumetric computed tomography images from a single projection view via deep learning. Nat. Biomed. Eng. *3*, 880–888.
21. Kasten, Y., Doktofsky, D., and Kovler, I. (2020). End-To-End Convolutional Neural Network for 3D Reconstruction of Knee Bones from Bi-planar X-Ray Images (Springer International Publishing), pp. 123–133.
22. Wang, L., Liang, D., Yin, X., Qiu, J., Yang, Z., Xing, J., Dong, J., and Ma, Z. (2020). Weakly-supervised 3D coronary artery reconstruction from two-view angiographic images. Preprint at arXiv. https://doi.org/10.48550/arXiv.2003.11846.
23. Rumelhart, D.E., Hinton, G.E., and Williams, R.J. (1986). Learning representations by back-propagating errors. Nature (London) *323*, 533–536.
24. LeCun, Y., Boser, B., Denker, J.S., Henderson, D., Howard, R.E., Hubbard, W., and Jackel, L.D. (1989). Backpropagation applied to handwritten zip code recognition. Neural Comput. *1*, 541–551.
25. Falk, T., Mai, D., Bensch, R., Çiçek, Ö., Abdulkadir, A., Marrakchi, Y., Böhm, A., Deubner, J., Jäckel, Z., Seiwald, K., et al. (2019). U-Net: deep learning for cell counting, detection, and morphometry. Nat. Methods *16*, 67–70.
26. Wang, Z., Bovik, A.C., Sheikh, H.R., and Simoncelli, E.P. (2004). Image quality assessment: from error visibility to structural similarity. IEEE Trans. Image Process. *13*, 600–612.
27. Greve, T., Sollmann, N., Hock, A., Hey, S., Gnanaprakasam, V., Nijenhuis, M., Zimmer, C., and Kirschke, J.S. (2020). Highly accelerated time-of-flight magnetic resonance angiography using spiral imaging improves conspicuity of intracranial arterial branches while reducing scan time. Eur. Radiol. *30*, 855–865.
28. Lell, M.M., Kramer, M., Klotz, E., Villablanca, P., and Ruehm, S.G. (2009). Carotid computed tomography angiography with automated bone suppression: a comparative study between dual energy and bone subtraction techniques. Invest. Radiol. *44*, 322–328.
29. Anxionnat, R., Bracard, S., Ducrocq, X., Trousset, Y., Launay, L., Kerrien, E., Braun, M., Vaillant, R., Scomazzoni, F., Lebedinsky, A., and Picard, L. (2001). Intracranial aneurysms: clinical value of 3D digital subtraction angiography in the therapeutic decision and endovascular treatment. Radiology *218*, 799–808.
30. Kucukay, F., Okten, R.S., Tekiner, A., Dagli, M., Gocek, C., Bayar, M.A., and Cumhur, T. (2012). Three-dimensional volume rendering digital subtraction angiography in comparison with two-dimensional digital subtraction angiography and rotational angiography for detecting aneurysms and their morphological properties in patients with subarachnoid hemorrhage. Eur. J. Radiol. *81*, 2794–2800.
31. Sarder, P., and Nehorai, A. (2006). Deconvolution methods for 3-D fluorescence microscopy images. IEEE Signal Process. Mag. *23*, 32–45.
32. Jing, L., and Tian, Y. (2021). Self-supervised visual feature learning with deep neural networks: a survey. IEEE Trans. Pattern Anal. Mach. Intell. *43*, 4037–4058.
33. Li, X., Zhang, G., Wu, J., Zhang, Y., Zhao, Z., Lin, X., Qiao, H., Xie, H., Wang, H., Fang, L., and Dai, Q. (2021). Reinforcing neuron extraction and spike inference in calcium imaging using deep self-supervised denoising. Nat. Methods *18*, 1395–1400.
34. Xie, Y., Xu, Z., Zhang, J., Wang, Z., and Ji, S. (2021). Self-supervised learning of graph neural networks: a unified review. Preprint at arXiv. https://doi.org/10.48550/arXiv.2102.10757.
35. Paszke, A., Gross, S., Chintala, S., Chanan, G., Yang, E., DeVito, Z., Lin, Z., Desmaison, A., Antiga, L., and Lerer, A. (2017). Automatic Differentiation in PyTorch.
36. Kingma, D.P., and Ba, J. (2017). Adam: a method for stochastic optimization. Preprint at arXiv. https://doi.org/10.48550/arXiv.1412.6980.
37. Matthew. (2012). ADADELTA: an adaptive learning rate method. Preprint at arXiv. https://doi.org/10.48550/arXiv.1212.5701.
38. Malhotra, A., Wu, X., Forman, H.P., Matouk, C.C., Gandhi, D., and Sanelli, P. (2018). Management of tiny unruptured intracranial aneurysms. JAMA Neurol. *75*, 27–34.

STAR★METHODS

KEY RESOURCES TABLE

REAGENT or RESOURCE	SOURCE	IDENTIFIER
Critical commercial assays		
DSA equipment 1	Siemens Medical Systems	Axiom Artis Zee
DSA equipment 2	General Electric Healthcare	Innova IGS 540
DSA equipment 3	Siemens Medical Systems	Axiom Artis Q Biplane
Software and algorithms		
Python 3.9	Python	https://www.python.org
PyTorch 1.7.1	PyTorch	https://pytorch.org
Matlab R2017a	Matlab	https://ww2.mathworks.cn
Imaris 7.4.2	Imaris	https://imaris.oxinst.com/
R 4.0.5	R	https://www.r-project.org/
SPSS 25.0	SPSS	https://spss.en.softonic.com/
SSDR code	This paper	https://github.com/zhouzhenghong-gt/

RESOURCE AVAILABILITY

Lead contact

Further information and requests for resources should be directed to and will be fulfilled by the lead contact, Chuansheng Zheng (hqzcsxh@sina.com).

Materials availability

This study did not generate new unique reagents.

Data and code availability

The relevant data supporting the results reported herein are available within the text. The code used to perform the analyses and produce figures can be found on GitHub: https://github.com/zhouzhenghong-gt/self-supervised-3D-DSA-reconstructio-network#self-supervised-learning-enables-excellent-3d-digital-subtraction-angiography-reconstruction-from-ultra-sparse-2d-projection-views. The raw datasets are protected because of patient privacy. Any additional information required to reanalyze the data reported in this work paper is available from the lead contact upon request.

EXPERIMENTAL MODEL AND SUBJECT DETAILS

This study involved human subjects, and the criteria are described in the Introduction section and Figure S1. This study retrospectively enrolled patients from three Chinese hospitals (Institution I: Wuhan Union Hospital, Institution II: Wuhan Union West Hospital, Institution III: Hubei Provincial People's Hospital). This study has been reviewed and approved by the institutional review boards of the Medical Ethics Committee of the Union Hospital, Tongji Medical College, Huazhong University of Science and Technology. The requirement of informed consent was waived. All the centers registered and approved the studies labeled as Project Number 2022 (0311) and this study.

METHOD DETAILS

Overall description of the scientific research problem

It is important to reconstruct the 3D image of human cerebral vasculature from 2D-DSA images. Traditional non-DL 3D medical reconstruction methods require the use of a large number of 2D-DSA images, resulting in the use of a large amount of radiation. The end-to-end optimization method cannot be followed under these conditions. The use of the DL method for 3D medical reconstruction is hindered by the fact that it is difficult to accurately obtain true 3D labels. The gold standard method requires the use of a large number of 2D images. Complex image processing and matching procedures should be conducted to obtain a good visualization effect for the obtained 3D image. But, it is different from that of the real 3D label as the traditional methods inevitably result in vascular distortions.[31] The traditional methods are used as the gold standard under sub-optimal conditions.

To solve this problem, we propose a novel self-supervised 3D medical image reconstruction method that can be used without accessing the true 3D labels. The method can be used to train the 3D reconstruction network using 2D images and camera angles. This method is different from other self-supervised DL methods.[32–34] Pretext tasks for pre-training need not be set up in this case, and the method is different from the classic autoencoder self-supervised method. It is used as the input itself for supervision to achieve self-supervised learning.

Study design

To test the reconstruction results under varying sparse sampling conditions, 4/6/8/10/12 projections from multi-view 2D perspectives were used as the inputs, and the required projection angles are shown in Table S1. 3D images reconstructed using traditional methods were obtained and used as the gold standard. PSNR/MSE/MAE/SSIM values were used as the objective quantitative indicators, and the scores recorded by the radiologists were used as the subjective indicators to evaluate the reconstruction effect of the SSDR network. This retrospective study has been reviewed and approved by the institutional review boards of the Medical Ethics Committee of the Union Hospital, Tongji Medical College, and Huazhong University of Science and Technology. The requirement of informed consent was waived.

Detailed steps of data acquisition

Three DSA equipment (Axiom Artis Zee, Siemens Medical Systems, Erlangen, Germany; Innova IGS 540, General Electric Healthcare, United Kingdom; Axiom Artis Q Biplane, Siemens Medical Systems, Erlangen, Germany) from the Wuhan Union Hospital, Wuhan Union West Hospital, and Hubei Provincial People's Hospital, was used to record the 2D images. Iodixanol (Hengrui, Jiangsu, China; 320 mg/mL) was used as the contrast agent. A high-pressure injector (MEDRAD Mark VII ProVis (Bayer), Berlin, Germany) was used to inject iodixanol. The data to reconstruct the 3D images were collected under the 5 s DSA Head mode. During the process, the C-arm was rotated by 199.5° over 5 s. The contrast agent was injected into the body 1 s earlier before C-arm rotated, and the flow rate of the contrast agent was maintained at 3.5 mL/s. A total of 21 mL of the contrast agent was used for imaging once. A total of ~133 2D projection images were recorded, and the images were transferred to the workplace workstation for 3D reconstruction (gold standard). The institutional review board-approved protocols were followed to approach the patients who were being treated at the participating institutions, and the patients gave informed consent.

Problem description and response strategy

When a series of 2D projections $\{X_1, X_2 \ldots X_N\}$ and their corresponding view angles $\{\theta_1, \theta_2 \ldots \theta_N\}$ are known, a deep neural network (as the mapping function F) is used to predict the 3D reconstruction result (Y_{pred}). This problem can be formulated as follows:

$$F(X_1, X_2 \ldots X_N, \theta_1, \theta_2 \ldots \theta_N) = Y_{pred} \quad \text{(Equation 1)}$$

When the 2D projection images corresponding to different projection views enter the network in the form of different channels, the model sends as output the 3D prediction result of a single channel. Following this, the 3D prediction result is projected under conditions of the same view as the input. The calculation method of the projection can be expressed as follows:

$$Y_i = P(Y_{pred}; \theta_i), \quad \text{(Equation 2)}$$

where Y_i represents the of Y_{pred} at an angle θ_i, and i represents the number of views.

In an actual clinical setting, the electron beam is emitted in the form of a cone. The 2D-DSA images should be corrected and approximated to the projection of the parallel beam to facilitate the diagnosis process. Therefore, we used parallel beams from different angles for projection to simulate a real environment. The maximum value corresponding to each beam was used to obtain a digitally generated projection. This can be expressed as follows:

$$Y_i(r_p) = P(Y_{pred}(r); \theta_i) = \max(Y_{pred}(r)) \quad \text{(Equation 3)}$$

where r represents the position index of a beam passing through Y_{pred} at an angle of θ_i, Y_i is a 2D image projected by Y_{pred} at an angle of θ_i, r_p is the index of the corresponding position in Y_i after the projection of r at the angle of θ_i, and r is orthogonal to the plane where Y_i is located.

3D U-Net at full resolution can be used to consider less context information as a large single sample data corresponding to the 3D reconstruction task. Hence, we used the cascade method to increase the receptive field of the 3D reconstruction at high resolution and improve the results obtained using the 3D reconstruction method. As shown in the figure, we first trained the 3D U-Net on the down-sampled data and then used the second full-resolution 3D U-Net to refine the low-resolution reconstruction results. In clinical applications, the reconstruction resolution and the number of cascades can be determined according to application requirements.

The cascaded neural network can be mathematically expressed as follows:

$$F_1(X_1, X_2 \ldots X_N, \theta_1, \theta_2, \ldots \theta_N) = Y'_{pred} \quad \text{(Equation 4)}$$

$$F_2\left(X_1, X_2 \ldots X_N, \theta_1, \theta_2, \ldots \theta_N, Y'_{pred}\right) = Y''_{pred} \quad \text{(Equation 5)}$$

where F_1 represents the first-stage neural network, F_2 represents the second-stage neural network, Y'_{pred} represents the prediction result of the first-stage neural network, and Y''_{pred} is the prediction result of the second-stage neural network. If a two-stage cascade is considered, Y''_{pred} denotes the final output of the model. During training and testing, the input images are scaled to different resolutions using the linear interpolation method according to the network requirements at different stages.

Detailed description of the two stages of the SSDR network

In the dimensional ascending stage, different projections are input into different channels. We used 4/6/8/10/12 channels (according to the number of input projections) to conduct the studies. Two datasets of different scales were input into low- and high-resolution reconstruction stages to reconstruct high-quality multi-scale vasculatures. In the high-resolution scale, the image size was 512 × 395 pixels. In the low-resolution scale, the 512 × 395 pixels per channel were resized to 256 × 128 pixels per channel. Following this, under the given conditions of two different scales, the image was copied 512 (256) times along the vertical direction of each viewing angle, resulting in the generation of 3D data in each channel containing 512 × 512×395 (256 × 256×128) voxels. We used the 3D space formed under conditions of the 180° viewing angle to constrain data to ensure that multi-channel data can be integrated into the same channel. In other words, we cropped the part outside the 3D space formed under conditions of the 180° viewing angle to generate data in the same space. Following this, we extracted the features of each channel following the process of the convolution operation, and the obtained data consisted of 512 × 512×395 × 16 (256 × 256×128 × 16) voxels per channel. Finally, the low-resolution data obtained for each channel was introduced to obtain the final data (256 × 256×128 × 16 voxels). This was the input data of the low-resolution reconstruction stage. The input of the high-resolution data was cascaded with the output data of the low-resolution reconstruction stage, and the resultant data was used as an input during the high-resolution reconstruction stage. The whole process is presented in Figure 3. In the low-resolution reconstruction stage, the data are used for encoding and decoding. The data are then subjected to successive resizing and convolution operations. We used a 3D U-Net[25] for encoding and decoding. The processes of encoding and decoding and the changes in the data size are presented in Figure S5. During the encoding process, the data is subjected to four cycles of pooling and convolution operations (convolution kernel size: 3 × 3×3). During the decoding process, the data are subjected to four cycles of up-sampling and convolution operations (convolution kernel size: 3 × 3×3). The method of skip connection is used to connect the data of the same size obtained during the processes of encoding and decoding. Finally, 16 groups of data are integrated into 1 group of the 3D data following a 1 × 1×1 convolution operation. In the high-resolution reconstruction stage, the same 3D U-Net is used. The only difference lies in the fact that the input data is of higher resolution. The encoding and decoding processes and the changes in the data size are presented in Figure S6. At the end of the stage, a post-processing method was used to obtain the final 3D data. A detailed description of the post-processing method has been presented in Detailed description of the post-processing method and Figure S6. It is worth noting that a series of 2D projections $\{X_1, X_2, \ldots X_N\}$ are normalized in the interval of [0,1] before they are input into CNN. We cut the original data in the Z-direction because of the limitations of the graphics processing unit (GPU, 48GB memory) of our computer. The data were then imported for encoding. The patch size was 256 × 256×32 in the low-resolution reconstruction stage and 512 × 512×32 in the high-resolution reconstruction stage.

Training details

We trained CNN to predict the 3D images (Y_{pred}) with input images (X) containing a stacked sequence of 2D projections. We define the cost function as the MSE between the 2D projections of the 3D prediction and corresponding input 2D projections. The maximum intensity projection (MIP) of the reconstructed 3D-DSA was used as the 2D projections of the 3D prediction. The loss calculation function is expressed as follows:

$$\text{Loss} = \sum_{i=1}^{N} \|Y_i - X_i\|_2^2 = \sum_{i=1}^{N} \|P(Y_{pred}; \theta_i) - X_i\|_2^2, \quad \text{(Equation 6)}$$

where N is the number of views used in training.

The model was optimized iteratively following the method of stochastic gradient descent. We used the same training strategy and hyper-parameters for all experiments to objectively compare the reconstruction results obtained using different numbers of projections as input. We implemented the network using the PyTorch[35] library (with CUDA and CUDNN support). The adam optimizer[36] was used to minimize the loss function and update the network parameters iteratively following the process of back-propagation. The exponential decay learning rate[37] was used in all CNN-based experiments. The initial learning rate corresponding to the low- and high-resolution reconstruction stages was set to 0.001 and 0.03 (decay: 0.9), respectively. Finally, the best checkpoint model with the smallest validation loss was saved as the final model. We respectively trained the low- and high-resolution reconstruction stages network for 1,500 and 400 epochs using the Nvidia RTX 8000 graphics processing unit (GPU, 48GB memory).

Detailed description of the post-processing method

There must be a situation where a patch of 512 × 512×32 voxels is present in the background to ensure that all outputs are characterized by 512 × 512×395 voxels (in the test data). The intensities of the noise signals in such a patch are enhanced using the test model (Figure S6A; yellow arrows) if the post-processing method is not initiated at this point. We addressed this problem using the sparse reconstruction method to analyze the vasculature signal obtained from the 3D reconstruction result. It was observed that the average value of each patch voxel was less than 0.01 when the gold standard was used for analysis. Our model predicts that the average value of the background patch prediction results will exceed 0.01. We set the values corresponding to the patches to zero for cases where the average predicted result exceeded 0.01 during post-processing.

However, in some extreme cases (e.g., for the case of 4 projections), the predicted value corresponding to the dense blood vessels tends to be larger, resulting in inaccurate predictions (Figure S6B; red arrows). At this point, we can address the problem by tuning the threshold, that is, by increasing the value from 0.01 to 0.02. The adjusted result is shown in Figure S6C.

QUANTIFICATION AND STATISTICAL ANALYSIS

Subjective and objective evaluations

We used the trained model to analyze a testing dataset to evaluate the performance of the method. We analyzed the reconstruction results using both subjective and objective evaluation indicators. Furthermore, we classified lesions into three groups: tiny aneurysms (<3 mm), small aneurysms (3–7 mm), and "others" by measuring the long diameter of the aneurysms based on the standard criteria.[38] We considered the 3D reconstruction results obtained using the 8 projections as the typical in-depth analytical results of these three types of lesions.

Subjective evaluations: The quadratic weighted κ test was used to test the scoring consistency of the two readers (the scoring method used for evaluating the 3D reconstruction results and the diagnostic confidence were also studied). The strength of agreement between the observers was classified as poor ($\kappa < 0.2$), fair ($0.2 \le \kappa < 0.4$), moderate ($0.4 \le \kappa < 0.6$), strong ($0.6 \le \kappa < 0.8$), and super ($0.8 \le \kappa < 1.0$). The Friedman test was used to compare the image quality scores obtained using 4, 6, 8, 10, and 12 projections (Institution I). The Kruskal–Wallis test was conducted to compare the image quality scores obtained using the multicenter 8 projections method (scores were derived from the average score of R1 and R2). The Bonferroni method was used to correct the significance level, and the results were compared. The scores of image quality and diagnostic confidence were presented as mean ± SD. Pairwise comparisons between any two views were performed following a post hoc analysis method, following the process of Bonferroni correction. The different grades of aneurysms were analyzed. The Kruskal–Wallis test was conducted to compare the objective indices and the diagnostic confidence, and the quadratic weighted κ test was conducted to determine the consistency of the diagnostic confidence obtained by different observers. $p < 0.05$ was considered statistically significant.

Objective evaluations: Four key quantitative indicators (SSIM, MAE, MSE, and PSNR) were used to study the similarities and differences between our results and those obtained using the gold standard. MAE/MSE is the L1-norm/L2-norm error, SSIM score is used for measuring the overall similarity between the two images, and PSNR is defined as the ratio between the maximum signal power and the noise power that affects the image quality (it is widely used to determine the quality of the reconstructed image). In general, a lower value of MAE and MSE or a higher SSIM and PSNR score indicates a better reconstruction result.

$$\mathrm{MSE} = \|ref - rec\|_2^2 \quad \text{(Equation 7)}$$

$$\mathrm{MAE} = \|ref - rec\|_1^1 \quad \text{(Equation 8)}$$

$$\mathrm{PSNR} = 20\log_{10}\frac{\max(ref)\sqrt{N}}{\|ref - rec\|_2^2} \quad \text{(Equation 9)}$$

$$\mathrm{SSIM} = \frac{2\mu_{ref}\mu_{rec} + c_1}{\mu_{ref}^2 + \mu_{rec}^2 + c_1} \cdot \frac{2\sigma_{ref\_rec} + c_2}{\sigma_{ref}^2 + \sigma_{rec}^2 + c_2} \cdot \frac{\sigma_{ref\_rec} + c_3}{\sigma_{ref}\sigma_{rec} + c_3} \quad \text{(Equation 10)}$$

Here *rec* is the reconstructed image, *ref* denotes the reference image, and *N* is the total number of image voxels. The SSIM index is a multiplicative combination of the luminance term, the contrast term, and the structural term. The values of μ_{ref} *and* μ_{rec} *are* the mean values of reconstructed images and reference images, respectively. The values of σ_{ref} *and* σ_{rec} are the standard deviations of reconstructed images and reference images, respectively. The value of σ_{ref_rec} denotes the covariance of the reconstructed image and the reference image. The values of $c_1, c_2,$ *and* c_3 are the non-negative real numbers that specify the regularization constants for the luminance, contrast, and structural terms, respectively.

Evaluation of image quality and diagnostic confidence by radiologists

Before conducting the experiments, the details of the patients in the images were hidden. The process of subjective evaluation of the reconstructed images is divided into two aspects (analysis of image quality and diagnostic confidence for vascular pathology).

Two radiologists (R1 and R2) independently studied the reconstructed images. The anteroposterior and lateral images were used to evaluate the image quality. In particular, the overall image performance and the degree of vessel detectability were evaluated. The evaluation criteria are presented in Table S5. The 3D reconstruction results obtained using 4, 6, and 8 projections (for 10 random patients of Institution I) were used to determine the reproducibility of the scoring method. That is, a total of 30 sets were selected for inter-observer consistency analysis.

The 3D images reconstructed from 8 projections were used for evaluating the diagnostic confidence, and the gold standards were used to determine whether pathological vessels exist. R1 and R2 independently followed the multi-directional rotational viewing method to study the reconstructed 3D images using the Imaris software. They used the following scoring standards to determine the diagnostic confidence of the pathological and non-pathological vessels: 1 point = very uncertain; 2 points = not so sure; 3 points = neutral; 4 points = basically certain; 5 points = very sure. Points ≥ 4 are considered as high diagnostic confidence.

Reconstruction results with limited angle projections

We conducted an experiment with three types of inputs with eight projection views. The projection angles used by the three schemes cover 45°, 90°, and 180°. This experiment was conducted to verify if the reconstruction ability of 3D-DSA with limited angle projections would be affected. The reconstruction results obtained by analyzing the 102 cases reported by Institutions II and III were quantitatively analyzed using SSIM/MAE/MSE/PSNR. The statistical results are listed in Table S2. It can be seen from this table that the reconstruction results are significantly affected when limited angle projections are used as input.

Experiments to compare the data obtained by three different methods

We compared three types of reconstruction results: (1) Only stage 1 is used for reconstruction; (2) Only stage 2 is used for reconstruction; (3) Both two stages are used for reconstruction. The reconstruction images obtained from stage 1, stage 2, both two stages, and the gold standard are presented in Figure S5A–S5D). The quality of the image presented in Figure S5A is poor. An area containing dense blood vessels was enlarged (yellow, green, tomato-red, and blue dotted boxes). The microvessels presented in the tomato-red dashed box were similar to those presented in the blue dashed box. The reconstruction results obtained by analyzing 50 cases reported by Institution I were quantitatively analyzed using SSIM/MAE/MSE/PSNR. Statistical results are presented in Figure S5E–S5H. It can be seen that the SSIM and MAE values for these three cases are at the same level. Analysis of the MSE and PSNR indicators reveals that the quality of the reconstructed images obtained from stage 1, stage 2, and both two stages improve gradually. In summary, the results proved that the absence of any one of the stages results in the deterioration in the quality of the reconstruction results.

Cell Reports Medicine, Volume *3*

Supplemental information

Self-supervised learning enables 3D digital subtraction angiography reconstruction from ultra-sparse 2D projection views: A multicenter study

Huangxuan Zhao, Zhenghong Zhou, Feihong Wu, Dongqiao Xiang, Hui Zhao, Wei Zhang, Lin Li, Zhong Li, Jia Huang, Hongyao Hu, Chengbo Liu, Tao Wang, Wenyu Liu, Jinqiang Ma, Fan Yang, Xinggang Wang, and Chuansheng Zheng

Supplementary Figures

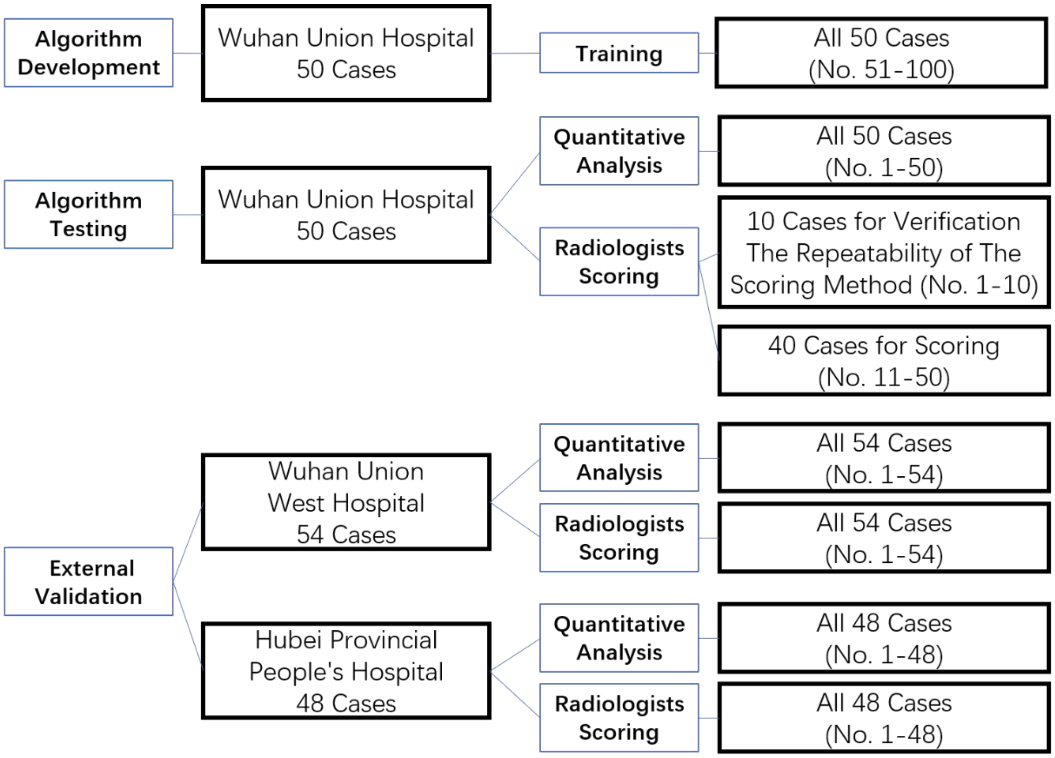

Figure S1. Flowchart outlining the data acquisition methods and the divisions made. Related to STAR METHODS.

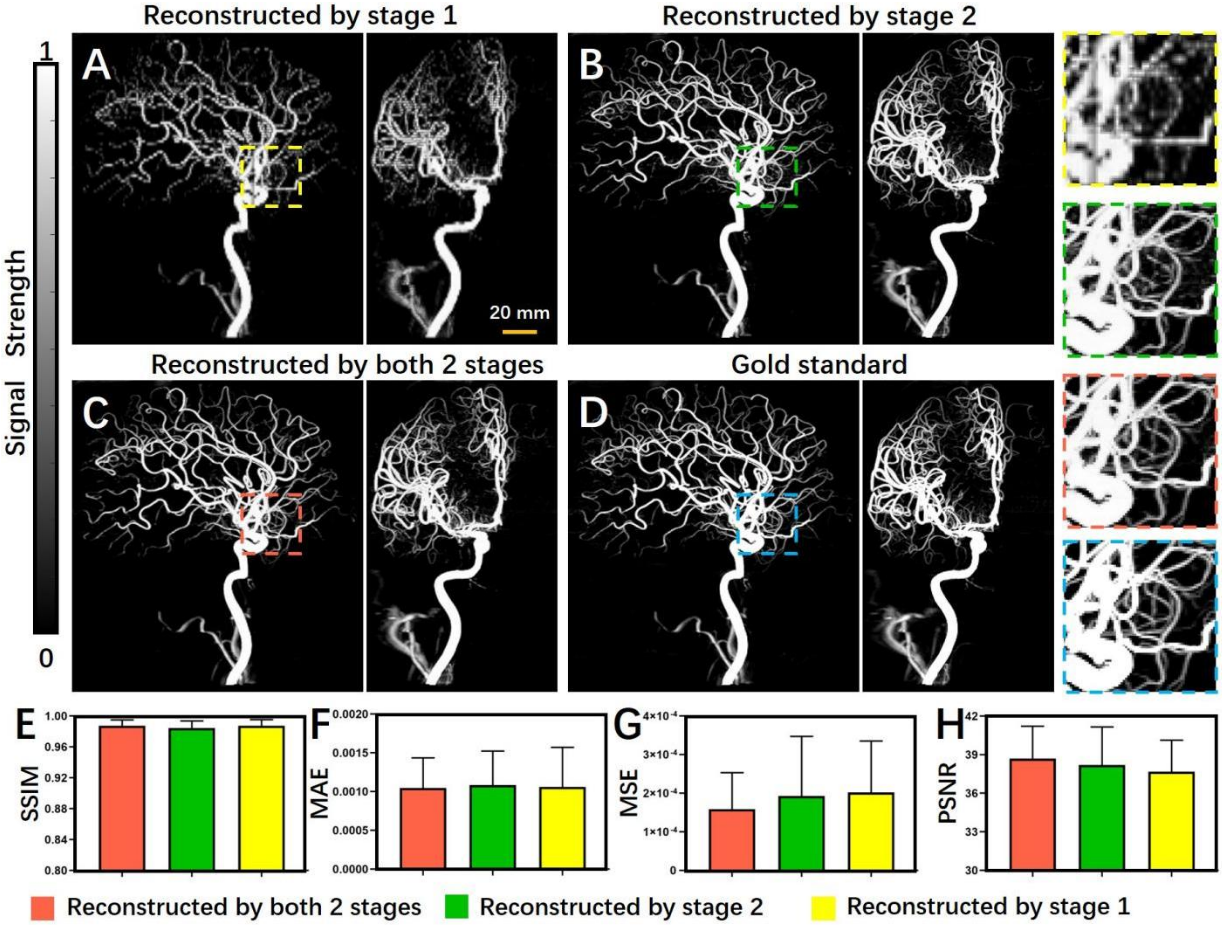

Figure S2. Comparison of reconstruction results. (A)–(D) Reconstruction images obtained from stage 1, stage 2, both 2 stages, and the gold standard. (E)–(H) Results obtained by quantitatively evaluating 50 cases of Institution I using SSIM/MAE/MSE/PSNR. Related to STAR METHODS.

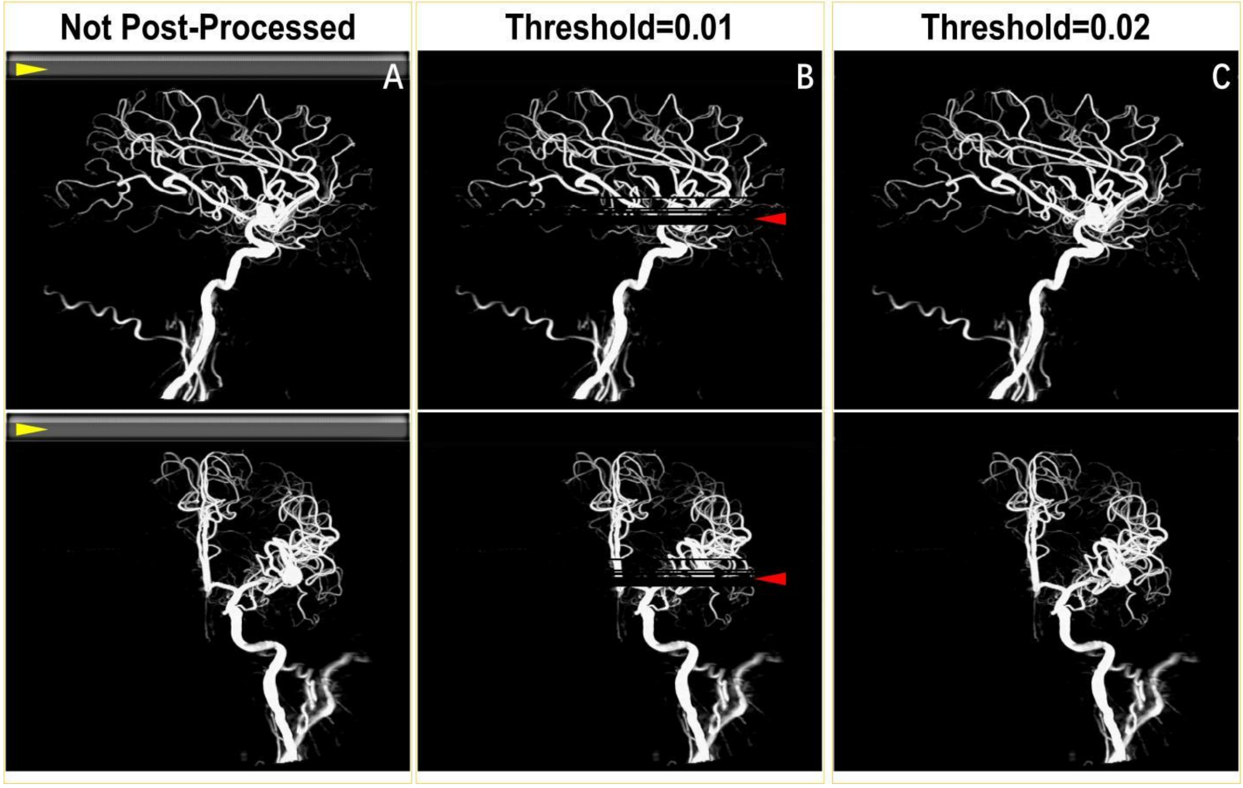

Figure S3. Reconstructed images. (A) Post-processed method not followed. (B) – (C) Post-processing method was followed (B. threshold=0.01 and C. threshold=0.02). Related to STAR METHODS.

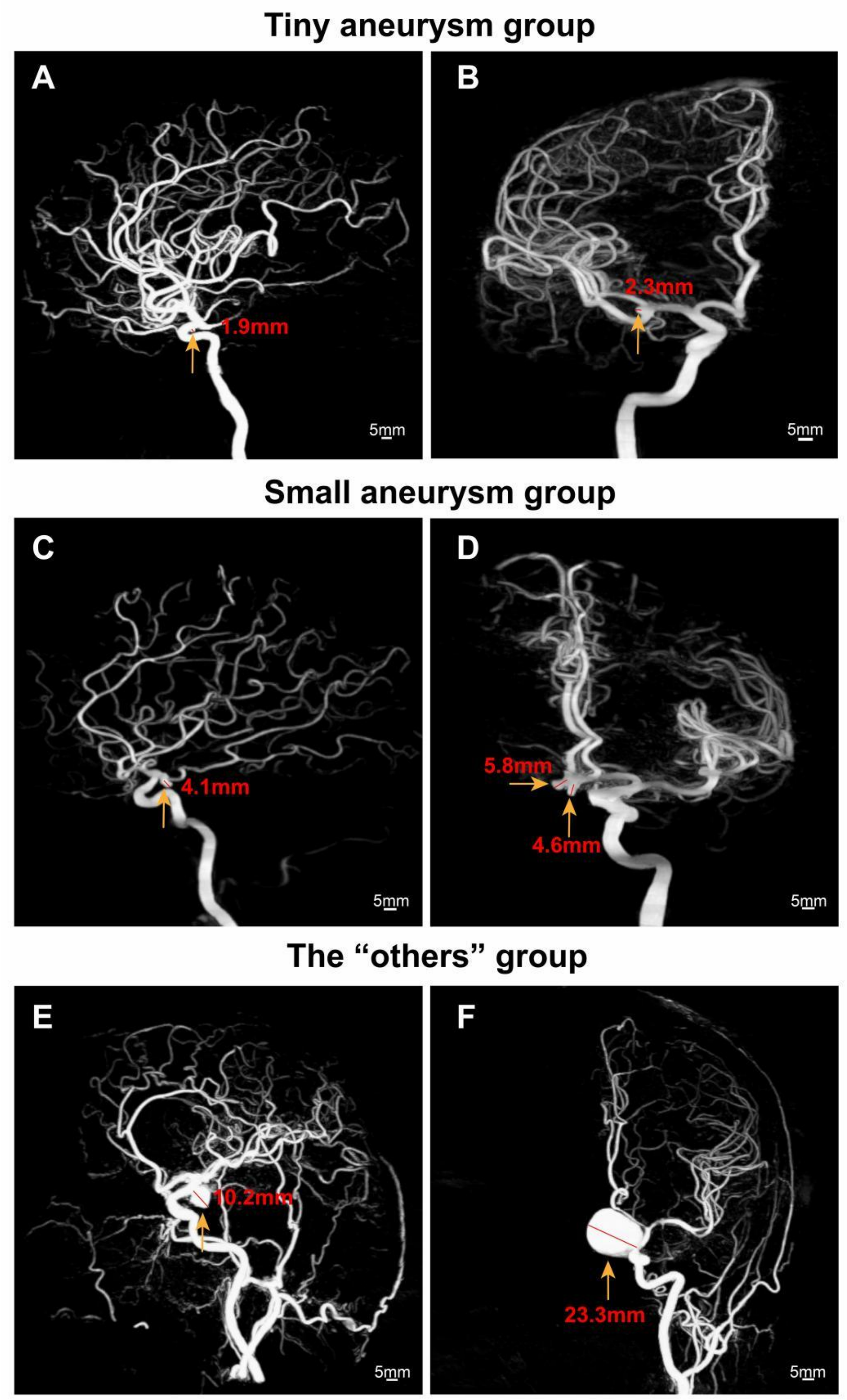

Figure S4. Random cases selected for presentation. (A) Tiny intracranial aneurysm (long diameter: 1.9 mm) located in the C6 segment. (B) Tiny aneurysm (long diameter: 2.3 mm) present at the bifurcation of the middle cerebral artery. (C) Small aneurysm (long

diameter: 4.1 mm) at the origin of the posterior cerebral artery. (D) Two aneurysms (long diameters: 5.8 mm and 4.6 mm, respectively) originating from the anterior communicating artery. (E) Significantly large intracranial aneurysm (long diameter: 10.2 mm) present in the C6 segment. (F) Large intracranial aneurysm (long diameter: 23.3 mm) originating from the C6 segment, and the A1 segment of the anterior cerebral artery was pushed upward. Related to STAR METHODS.

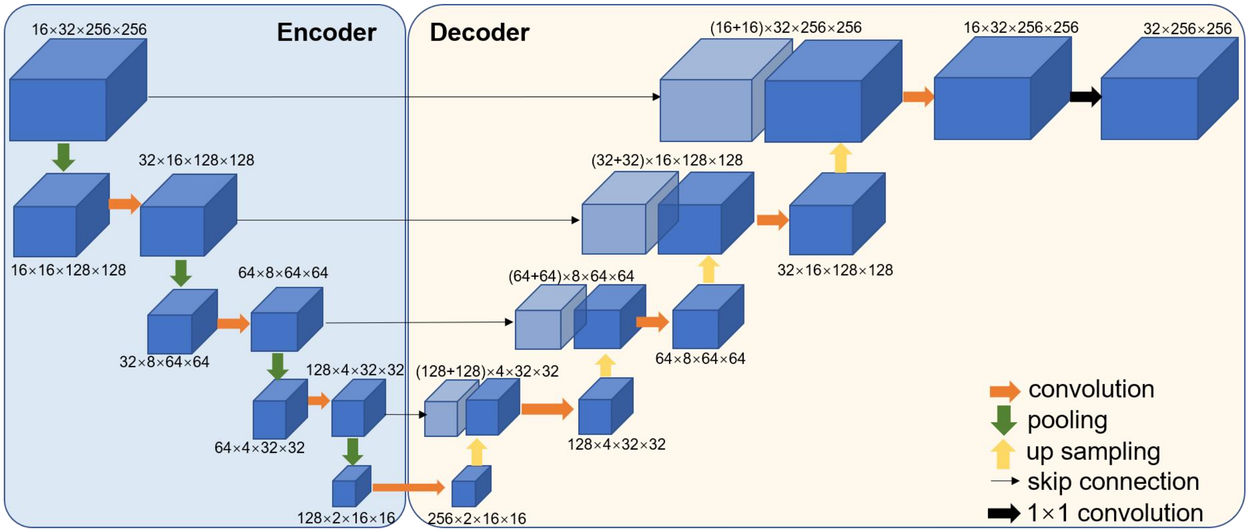

Figure S5. Encoding and decoding processes followed during the low-resolution reconstruction stage. Related to Figure 2.

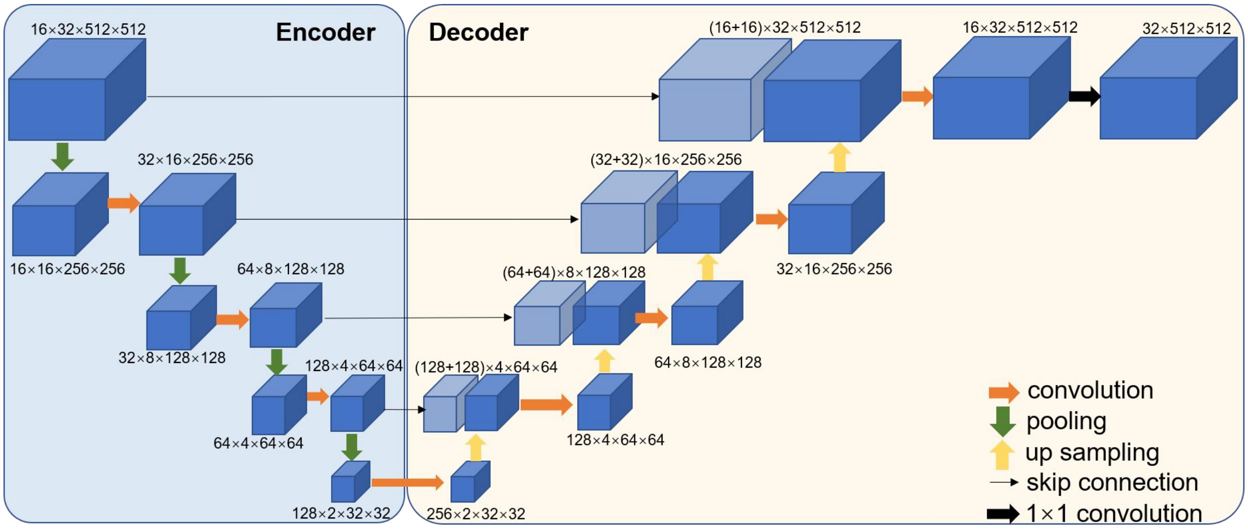

Figure S6. Encoding and decoding processes followed during the high-resolution reconstruction stage. Related to Figure 2.

Supplementary Tables

Table S1. Angles used for 4/6/8/10/12 projection views. Related to Figure 2.

Input Projections	Used Angles
4	45°/90°/135°/180°
6	30°/60°/90°/120°/150°/180°
8	22.5°/45°/67.5°/90°/112.5°/135°/157.5°/180°
10	18°/36°/54°/72°/90°/108°/126°/144°/162°/180°
12	15°/30°/45°/60°/75°/90°/105°/120°/135°/150°/165°/180°

Table S2. SSIM, PSNR, MSE, and MAE values corresponding to the reconstruction results under conditions of different cover angles of inputs with 8 projection views. Related to STAR METHODS.

Cover angles	SSIM	PSNR	MSE	MAE
45°	.969±.018	32.45±1.34	.000594±.000171	.002150±.000651
90°	.970±.018	35.17±1.59	.000324±.000113	.001714±.000610
180°	.982±.019	39.74±2.25	.000121±.000065	.000830±.000621

Table S3. SSIM, PSNR, MSE, and MAE values corresponding to the reconstruction results under conditions of different training data sizes. Related to STAR METHODS.

Training data sizes	SSIM	PSNR	MSE	MAE
50 cases	.982±.019	39.74±2.25	.000121±.000065	.000830±.000621
75 cases	.981±.016	40.21±2.17	.000108±.000057	.001211±.000500
100 cases	.980±.021	39.56±2.25	.000127±.000070	.001183±.000700

Table S4. SSIM/PSNR/MSE/MAE values corresponding to the multicenter 3D reconstruction images (4/6/8/10/12 projections were used). The best one is marked in red. Related to Figure 3 and Figure 4.

Institution	Input Projections	SSIM	PSNR	MSE	MAE
I	4	.954±.018	34.35±2.03	.000405±.000173	.002488±.000721
	6	.972±.014	37.49±2.42	.000206±.000111	.001771±.000524
	8	.987±.007	38.69±2.49	.000158±.000094	.001047±.000385
	10	.984±.008	39.30±2.54	.000139±.000085	.001078±.000453
	12	.984±.009	39.95±2.51	.000119±.000073	.001069±.000487
II	4	.959 ± .013	35.27 ± 1.56	.000314 ± .000095	.002160 ± .000427
	6	.977 ± .008	38.86 ± 1.76	.000140 ± .000052	.001472 ± .000270
	8	.989 ± .006	40.06 ± 1.66	.000106 ± .000038	.000855 ± .000223
	10	.987 ± .007	40.72 ± 1.59	.000090 ± .000032	.000843 ± .000249
	12	.988 ± .006	41.43 ± 1.55	.000077 ± .000027	.000805 ± .000239
III	4	.933 ± .022	36.55 ± 1.91	.000243 ± .000103	.002900 ± .000681
	6	.964 ± .022	39.35 ± 2.52	.000137 ± .000080	.001953 ± .000690
	8	.981 ± .016	39.58 ± 2.61	.000132 ± .000081	.001226 ± .000581
	10	.979 ± .017	39.88 ± 2.60	.000123 ± .000075	.001227 ± .000619
	12	.978 ± .017	40.33 ± 2.61	.000111 ± .000067	.001184 ± .000604

Table S5. Scoring method for evaluating 3D reconstruction results (ICA, internal carotid artery; ACA, anterior cerebral artery; MCA, middle cerebral artery). Related to STAR METHODS.

		Score					
		**0**	**1**	**2**	**3**	**4**	**5**
**The overall performance**		Gaps in the image	Severe noise	A lot of noise	Moderate noise	Slight noise	Invisible noise
**Vessel detectability**	**ICA**	Unable to evaluate	Difficulty in identifying vascular occlusion	Difficulty in identifying vascular occlusion stenosis	The vascular boundary of ICA is not clear, but it can be used to identify vascular stenosis	The vascular boundary of ICA is clear, and artifacts exist	The vascular boundary of ICA is clear and devoid of artifacts
	**ACA**	Unclear/not distinguishable	Only the A1 segment is distinguishable	A1 and A2 segments are distinguishable	A1, A2, and A3 segments are distinguishable	/	/
	**MCA**	Unclear/not distinguishable	Only the M1 segment is distinguishable	M1 and M2 segments are distinguishable	M1, M2, and M3 segments are distinguishable	/	/

Table S6. Evaluation results obtained by the two radiologists during the analysis of 30 sets of data. Related to STAR METHODS.

No.	The overall performance		ICA detectability		ACA detectability		MCA detectability	
	R1	R2	R1	R2	R1	R2	R1	R2
**4 Projections**								
**1**	0	0	3	4	0	0	0	0
**2**	0	0	3	3	0	0	0	0
**3**	0	0	4	4	0	0	0	0
**4**	0	0	3	2	3	3	3	3
**5**	3	1	3	3	3	3	3	3
**6**	1	2	3	2	3	3	3	3
**7**	3	3	4	4	3	3	3	3
**8**	0	0	4	4	0	0	0	0
**9**	0	0	4	3	0	0	0	0
**10**	3	3	4	4	3	3	3	3
**6 Projections**								
**1**	0	0	4	4	3	0	3	0
**2**	0	0	4	4	0	0	0	0
**3**	3	1	4	4	3	3	3	3
**4**	3	3	4	3	3	3	3	3
**5**	4	3	4	3	3	3	3	3
**6**	3	3	4	3	3	3	3	3
**7**	4	4	4	4	3	3	3	3
**8**	0	0	4	4	3	3	3	3
**9**	3	2	4	4	3	3	3	3
**10**	4	4	5	4	3	3	3	3
**8 Projections**								
**1**	5	3	5	4	3	3	3	3
**2**	0	2	5	4	3	3	3	3

**3**	4	4	5	4	3	3	3	3
**4**	5	4	5	4	3	3	3	3
**5**	5	3	4	3	3	3	3	3
**6**	5	4	4	4	3	3	3	3
**7**	5	4	5	4	3	3	3	3
**8**	4	3	5	4	3	3	3	3
**9**	3	4	5	4	3	3	3	3
**10**	5	4	5	4	3	3	3	3

Table S7. Scores of the overall image performance obtained by the two radiologists for 40 cases. Related to Table 1.

No.	The overall performance									
	4 Projections		6 Projections		8 Projections		10 Projections		12 Projections	
	R1	R2	R1	R2	R1	R2	R1	R2	R1	R2
**11**	0	0	0	0	5	5	5	5	5	5
**12**	0	0	0	0	0	0	4	4	4	4
**13**	0	0	3	3	4	4	4	4	5	5
**14**	0	0	3	3	5	5	5	5	5	5
**15**	3	3	4	4	5	5	5	5	5	5
**16**	1	1	3	3	5	5	5	5	5	5
**17**	3	3	4	4	5	5	5	5	5	5
**18**	0	0	0	0	4	4	5	5	5	5
**19**	0	0	3	3	3	3	5	4	5	5
**20**	3	3	4	4	5	5	5	5	5	5
**21**	1	2	2	3	3	4	4	4	5	5
**22**	0	0	3	4	4	4	5	5	5	5
**23**	0	0	0	0	4	4	5	4	5	5
**24**	3	3	2	4	4	4	4	5	5	5
**25**	3	3	4	4	5	5	5	5	5	5
**26**	2	3	3	4	4	5	5	5	5	5
**27**	3	3	4	4	5	5	5	5	5	5
**28**	0	0	2	4	4	4	4	5	5	5
**29**	0	0	0	0	4	4	4	4	5	5
**30**	0	0	1	3	3	4	4	5	5	5
**31**	1	3	3	4	4	5	5	5	5	5
**32**	4	4	4	4	5	5	5	5	5	5
**33**	0	0	0	0	0	0	1	1	3	3
**34**	3	3	3	4	4	4	4	5	5	5
**35**	0	0	3	4	5	5	5	5	5	5
**36**	0	0	0	0	4	4	5	5	5	5

**37**	0	0	3	3	4	4	5	5	5	5
**38**	0	0	0	0	3	3	3	3	4	4
**39**	3	4	4	4	5	4	5	5	5	5
**40**	0	0	3	3	4	4	4	4	5	5
**41**	3	3	4	4	5	5	5	5	5	5
**42**	2	3	4	4	0	5	5	5	5	5
**43**	3	3	3	4	5	5	5	5	5	5
**44**	0	0	0	0	4	4	5	5	5	5
**45**	0	0	0	0	4	4	5	5	5	5
**46**	0	0	3	4	4	5	5	5	5	5
**47**	4	3	5	5	5	5	5	5	5	5
**48**	0	0	3	4	4	4	4	5	5	5
**49**	4	4	4	4	5	5	5	5	5	5
**50**	0	0	3	4	5	4	5	5	5	5

Table S8. Scores obtained by the two radiologists for ICA detectability for 40 data. Related to Table 1.

No.	The ICA detectability									
	4 Projections		6 Projections		8 Projections		10 Projections		12 Projections	
	R1	R2	R1	R2	R1	R2	R1	R2	R1	R2
**11**	3	3	4	4	5	5	5	5	5	5
**12**	3	3	4	4	5	5	5	5	5	5
**13**	4	4	4	4	5	5	5	5	5	5
**14**	3	3	4	4	5	5	5	5	5	5
**15**	3	3	4	4	4	4	5	5	5	5
**16**	3	3	4	4	4	4	4	4	5	5
**17**	4	4	4	4	5	5	5	5	5	5
**18**	4	4	4	4	5	5	5	5	5	5
**19**	4	4	4	4	5	5	5	5	5	5
**20**	4	4	5	5	5	5	5	5	5	5
**21**	3	4	4	5	4	5	5	5	5	5
**22**	2	4	4	5	5	5	5	5	5	5
**23**	4	4	4	5	4	5	5	5	5	5
**24**	4	4	5	5	5	5	5	5	5	5
**25**	4	4	4	5	5	5	5	5	5	5
**26**	3	4	4	5	5	5	5	5	5	5
**27**	4	4	4	4	5	5	5	5	5	5
**28**	4	5	4	5	4	5	5	5	5	5
**29**	0	0	2	3	4	5	5	5	5	5
**30**	1	4	4	5	4	5	5	5	5	5
**31**	3	4	4	5	4	5	5	5	5	5
**32**	4	4	5	5	5	5	5	5	5	5
**33**	0	0	1	1	2	2	4	4	4	4
**34**	4	4	5	5	5	5	5	5	5	5
**35**	4	4	4	5	5	5	5	5	5	5
**36**	0	0	0	0	5	5	5	5	5	5

**37**	3	4	4	4	5	5	5	5	5	5
**38**	0	0	4	4	4	4	5	5	5	5
**39**	4	4	4	4	5	4	5	5	5	5
**40**	1	1	4	4	5	5	5	5	5	5
**41**	2	4	5	5	5	5	5	5	5	5
**42**	3	4	4	5	5	5	5	5	5	5
**43**	4	4	4	5	5	5	5	5	5	5
**44**	0	0	3	3	5	5	5	5	5	5
**45**	0	0	0	0	5	5	5	5	5	5
**46**	0	4	4	5	5	5	5	5	5	5
**47**	4	4	5	5	5	5	5	5	5	5
**48**	3	4	3	5	5	5	5	5	5	5
**49**	4	4	4	5	5	5	5	5	5	5
**50**	3	4	4	4	5	5	5	5	5	5

Table S9. Scores obtained by the two radiologists for ACA detectability for 40 data. Related to Table 1.

No.	The ACA detectability									
	4 Projections		6 Projections		8 Projections		10 Projections		12 Projections	
	R1	R2	R1	R2	R1	R2	R1	R2	R1	R2
**11**	0	0	3	3	3	3	3	3	3	3
**12**	0	0	0	0	3	3	3	3	3	3
**13**	0	0	3	3	3	3	3	3	3	3
**14**	3	3	3	3	3	3	3	3	3	3
**15**	3	3	3	3	3	3	3	3	3	3
**16**	3	3	3	3	3	3	3	3	3	3
**17**	3	3	3	3	3	3	3	3	3	3
**18**	0	0	3	3	3	3	3	3	3	3
**19**	0	0	3	3	3	3	3	3	3	3
**20**	3	3	3	3	3	3	3	3	3	3
**21**	3	3	3	3	3	3	3	3	3	3
**22**	0	0	3	3	3	3	3	3	3	3
**23**	0	0	0	0	3	3	3	3	3	3
**24**	3	3	3	3	3	3	3	3	3	3
**25**	3	3	3	3	3	3	3	3	3	3
**26**	3	3	3	3	3	3	3	3	3	3
**27**	3	3	3	3	3	3	3	3	3	3
**28**	3	3	3	3	3	3	3	3	3	3
**29**	0	0	0	0	3	3	3	3	3	3
**30**	0	0	3	3	3	3	3	3	3	3
**31**	3	3	3	3	3	3	3	3	3	3
**32**	3	3	3	3	3	3	3	3	3	3
**33**	0	0	0	0	0	0	3	3	3	3
**34**	3	3	3	3	3	3	3	3	3	3
**35**	3	3	3	3	3	3	3	3	3	3
**36**	0	0	0	0	3	3	3	3	3	3

**37**	0	0	3	3	3	3	3	3	3	3
**38**	0	0	3	3	3	3	3	3	3	3
**39**	3	3	3	3	3	3	3	3	3	3
**40**	0	0	3	3	3	3	3	3	3	3
**41**	3	3	3	3	3	3	3	3	3	3
**42**	3	3	3	3	3	3	3	3	3	3
**43**	3	3	3	3	3	3	3	3	3	3
**44**	0	0	0	0	3	3	3	3	3	3
**45**	0	0	0	0	3	3	3	3	3	3
**46**	0	0	3	3	3	3	3	3	3	3
**47**	2	2	3	3	3	3	3	3	3	3
**48**	0	0	3	3	3	3	3	3	3	3
**49**	3	3	3	3	3	3	3	3	3	3
**50**	0	0	3	3	3	3	3	3	3	3

Table S10. Scores obtained by the two radiologists for MCA detectability for 40 data. Related to Table 1.

No.	The MCA detectability									
	4 Projections		6 Projections		8 Projections		10 Projections		12 Projections	
	R1	R2	R1	R2	R1	R2	R1	R2	R1	R2
**11**	0	0	3	3	3	3	3	3	3	3
**12**	0	0	0	0	3	3	3	3	3	3
**13**	0	0	3	3	3	3	3	3	3	3
**14**	3	3	3	3	3	3	3	3	3	3
**15**	3	3	3	3	3	3	3	3	3	3
**16**	3	3	3	3	3	3	3	3	3	3
**17**	3	3	3	3	3	3	3	3	3	3
**18**	0	0	3	3	3	3	3	3	3	3
**19**	0	0	3	3	3	3	3	3	3	3
**20**	3	3	3	3	3	3	3	3	3	3
**21**	3	3	3	3	3	3	3	3	3	3
**22**	0	0	3	3	3	3	3	3	3	3
**23**	0	0	0	0	3	3	3	3	3	3
**24**	2	3	3	3	3	3	3	3	3	3
**25**	3	3	3	3	3	3	3	3	3	3
**26**	3	3	3	3	3	3	3	3	3	3
**27**	3	3	3	3	3	3	3	3	3	3
**28**	3	3	3	3	3	3	3	3	3	3
**29**	0	0	0	0	3	3	3	3	3	3
**30**	0	0	3	3	3	3	3	3	3	3
**31**	3	3	3	3	3	3	3	3	3	3
**32**	2	3	3	3	3	3	3	3	3	3
**33**	0	0	0	0	0	0	3	3	3	3
**34**	2	3	3	3	3	3	3	3	3	3
**35**	2	3	3	3	3	3	3	3	3	3
**36**	0	0	0	0	3	3	3	3	3	3

**37**	0	0	2	3	3	3	3	3	3	3
**38**	0	0	3	3	3	3	3	3	3	3
**39**	2	0	3	0	3	3	3	3	3	3
**40**	0	0	3	3	3	3	3	3	3	3
**41**	3	3	3	3	3	3	3	3	3	3
**42**	3	3	3	3	3	3	3	3	3	3
**43**	3	3	3	3	3	3	3	3	3	3
**44**	0	0	0	0	3	3	3	3	3	3
**45**	0	0	0	0	3	3	3	3	3	3
**46**	0	0	3	3	3	3	3	3	3	3
**47**	2	2	3	3	3	3	3	3	3	3
**48**	0	0	3	3	3	3	3	3	3	3
**49**	3	3	3	3	3	3	3	3	3	3
**50**	0	0	3	3	3	3	3	3	3	3

Table S11. Total scores obtained by the two radiologists for 40 data. Related to Table 1.

**No.**	**Total scores**									
	**4 Projections**		**6 Projections**		**8 Projections**		**10 Projections**		**12 Projections**	
	R1	R2	R1	R2	R1	R2	R1	R2	R1	R2
**11**	3	3	10	10	16	16	16	16	16	16
**12**	3	3	4	4	11	11	15	15	15	15
**13**	4	4	13	13	15	15	15	15	16	16
**14**	9	9	13	13	16	16	16	16	16	16
**15**	12	12	14	14	15	15	16	16	16	16
**16**	10	10	13	13	15	15	15	15	16	16
**17**	13	13	14	14	16	16	16	16	16	16
**18**	4	4	10	10	15	15	16	16	16	16
**19**	4	4	13	13	14	14	16	15	16	16
**20**	13	13	15	15	16	16	16	16	16	16
**21**	10	12	12	14	13	15	15	15	16	16
**22**	2	4	13	15	15	15	16	16	16	16
**23**	4	4	4	5	14	15	16	15	16	16
**24**	12	13	13	15	15	15	15	16	16	16
**25**	13	13	14	15	16	16	16	16	16	16
**26**	11	13	13	15	15	16	16	16	16	16
**27**	13	13	14	14	16	16	16	16	16	16
**28**	10	11	12	15	14	15	15	16	16	16
**29**	0	0	2	3	14	15	15	15	16	16
**30**	1	4	11	14	13	15	15	16	16	16
**31**	10	13	13	15	14	16	16	16	16	16
**32**	13	14	15	15	16	16	16	16	16	16
**33**	0	0	1	1	2	2	11	11	13	13
**34**	12	13	14	15	15	15	15	16	16	16
**35**	9	10	13	15	16	16	16	16	16	16
**36**	0	0	0	0	15	15	16	16	16	16

**37**	3	4	12	13	15	15	16	16	16	16
**38**	0	0	10	10	13	13	14	14	15	15
**39**	12	11	14	11	16	14	16	16	16	16
**40**	1	1	13	13	15	15	15	15	16	16
**41**	11	13	15	15	16	16	16	16	16	16
**42**	11	13	14	15	11	16	16	16	16	16
**43**	13	13	13	15	16	16	16	16	16	16
**44**	0	0	3	3	15	15	16	16	16	16
**45**	0	0	0	0	15	15	16	16	16	16
**46**	0	4	13	15	15	16	16	16	16	16
**47**	12	11	16	16	16	16	16	16	16	16
**48**	3	4	12	15	15	15	15	16	16	16
**49**	14	14	14	15	16	16	16	16	16	16
**50**	3	4	13	14	16	15	16	16	16	16

Table S12. (In Supplementary Excel) Scores obtained by two radiologists to analyze the overall image performance and vessel detectability using the 8 projection-method from external centers. Related to Table 2.

Table S13. (In Supplementary Excel) Diagnosis confidences obtained by two radiologists for reconstructed images from multicenter. Related to Table 3.

Table S14. Comparison of objective and subjective evaluation results obtained at different aneurysm stages. Data are presented as mean ± SD. Related to STAR METHODS.

	**Tiny (＜3mm, n=21)**	**Small (3~7mm, n=37)**	**Others (n=24)**	**p value [a]**
**Objective evaluation**				
**MAE**	.001104±.000450	.001050±.000446	.000894±.000259	0.16
**MSE**	.000136±.000069	.000124±.000057	.000114±.000062	0.46
**PSNR**	39.24±2.35	39.50±2.08	39.88±2.01	0.46
**SSIM**	.984±.011	.9849±.0135	.9895±.0043	0.19
**Subjective evaluation**				
**Reader 1**	4.17±0.89	4.20±0.90	4.33±0.92	0.70
**Reader 2**	4.17±0.78	4.09±0.89	4.25±0.90	0.71
**κ**	0.87	0.93	0.95	/
**p value [b]**	<0.001	<0.001	<0.001	/

a). The Kruskal–Wallis test was conducted to compare the objective indexes and the diagnostic confidence recorded by the same observer during the diagnosis of different aneurysm stages.

b). The quadratic weighted κ test was conducted to analyze the consistency of the diagnostic confidence of different observers at different stages of aneurysms.
